# A new brachylophosaurin (Dinosauria: Hadrosauridae) from the Upper Cretaceous Menefee Formation of New Mexico

**DOI:** 10.7717/peerj.11084

**Published:** 2021-04-02

**Authors:** Andrew T. McDonald, Douglas G. Wolfe, Elizabeth A. Freedman Fowler, Terry A. Gates

**Affiliations:** 1 Western Science Center, Hemet, CA, USA; 2 Zuni Dinosaur Institute for Geosciences, Springerville, AZ, USA; 3 Dickinson State University, Dickinson, ND, USA; 4 Department of Biological Sciences, North Carolina State University, Raleigh, NC, USA

**Keywords:** *Ornatops incantatus*, Brachylophosaurini, Hadrosauridae, Allison Member, Menefee Formation, New Mexico

## Abstract

Brachylophosaurini is a clade of hadrosaurid dinosaurs from the Campanian of western North America. Although well-known from northern localities in Montana and Alberta, including abundant material of *Brachylophosaurus canadensis* and *Maiasaura peeblesorum* and the holotypes of *Acristavus gagslarsoni* and *Probrachylophosaurus bergei*, material from southern localities in Utah and Colorado is restricted to a partial skull referred to *A*. *gagslarsoni* and several indeterminate specimens. Here we describe *Ornatops incantatus* gen. et sp. nov., a new brachylophosaurin known from a partial skeleton from the Allison Member of the Menefee Formation in New Mexico. *Ornatops* is the first brachylophosaurin reported from New Mexico and the southernmost occurrence of the clade. *Ornatops* shares with *Probrachylophosaurus* and *Brachylophosaurus* a caudally expanded nasofrontal suture on the frontals, but also exhibits an autapomorphic nasofrontal suture morphology, with a horizontal rostral region and elevated caudal region with two prominent parasagittal bumps, which is different from other brachylophosaurin specimens, including juvenile and adult *Brachylophosaurus*. A phylogenetic analysis places *Ornatops* in a trichotomy with *Probrachylophosaurus* and *Brachylophosaurus*, with *Maiasaura* and *Acristavus* as successive outgroups.

## Introduction

The Menefee Formation represents one of the most promising frontiers for exploring the early evolution of major dinosaur groups in Laramidia, the Upper Cretaceous landmass consisting of Mexico, the western United States, western Canada, and Alaska (Fig. 1 in Sampson et al. (2010)). Dating to approximately 84–78 million years ago (Siemers & King, 1974; Molenaar et al., 2002; Lucas et al., 2005), the Menefee Formation predates the most productive Upper Cretaceous dinosaur-bearing units in western North America, such as the Kirtland Formation of New Mexico, the Kaiparowits Formation of Utah, and the Dinosaur Park Formation of Alberta (Sullivan & Lucas, 2006; Jinnah et al., 2009; Fowler, 2017). Although widely exposed throughout the San Juan Basin of northwestern New Mexico, the Menefee Formation historically has produced only fragmentary dinosaur fossils (Hunt & Lucas, 1993), with the exception of a partial centrosaurine ceratopsid skeleton (Williamson, 1997).

Recent discoveries by a joint project conducted by the Western Science Center and Zuni Dinosaur Institute for Geosciences, assisted by volunteers from the Southwest Paleontological Society, have begun to illuminate the dinosaurs of the Allison Member of the Menefee Formation, starting with the new nodosaurid *Invictarx zephyri* (McDonald & Wolfe, 2018) and the new tyrannosaurid *Dynamoterror dynastes* (McDonald, Wolfe & Dooley, 2018) (additional tyrannosaurid material was recently reported by Dalman & Lucas (2018)). Here we describe a contemporaneous new hadrosaurid, known from a partial skeleton including the caudal region of the skull. This skeleton is the first hadrosaurid specimen from the Menefee Formation that is diagnostic to genus and species. In this article, we name the new taxon and describe the available cranial bones, which exhibit the pertinent diagnostic features. A forthcoming digital cranial endocast, the postcranial anatomy, and osteohistology of the holotype will be described in a future contribution (A.T. McDonald et al., 2021, in preparation).

**Figure 1:**
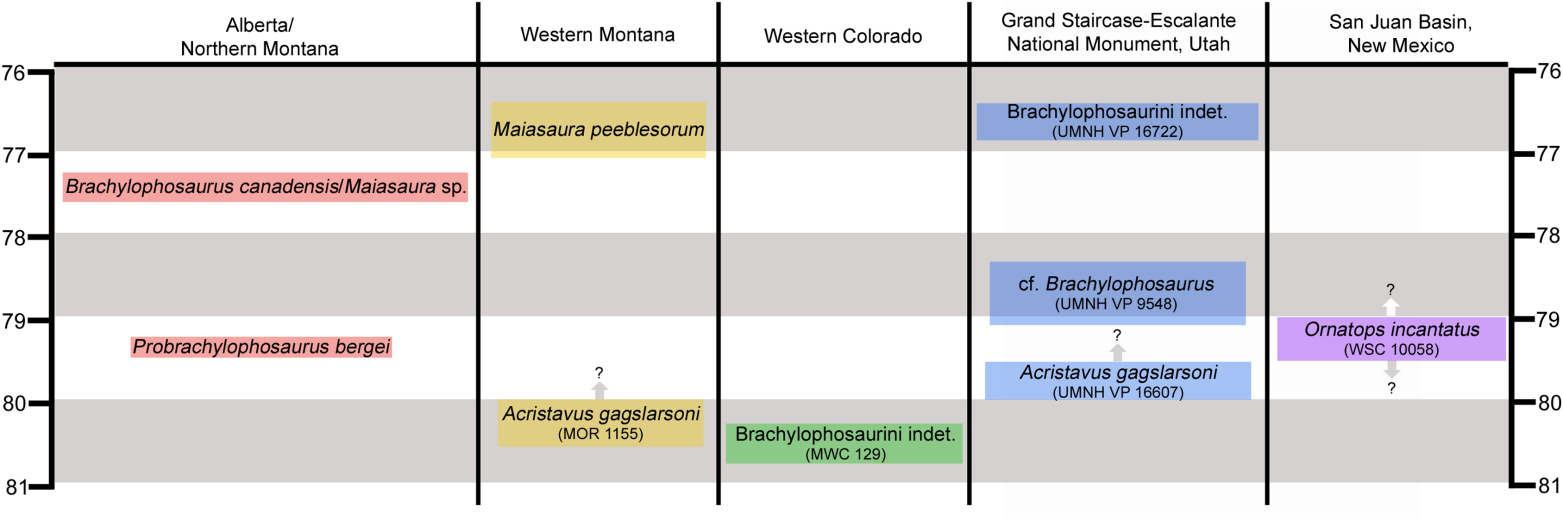
Chronostratigraphic and geographic distribution of Brachylophosaurini in Laramidia. Ages on the left and right are in millions of years. Occurrence data are from Lucas et al. (2006), Gates et al. (2011, 2013, 2014), Freedman Fowler & Horner (2015), Fowler (2017), and McFeeters et al. (2020). Height of colored text boxes denotes possible age ranges, with uncertainty in age ranges indicated by arrows and question marks.

Features of the skull roof and braincase identify the new hadrosaurid as a member of Brachylophosaurini (Gates et al., 2011; Freedman Fowler & Horner, 2015), one of several subclades within Saurolophinae, the “solid-crested” hadrosaurids (Prieto-Márquez, 2010a). Apart from the possible brachylophosaurin *Wulagasaurus dongi* from the Maastrichtian of China (Xing et al., 2012), brachylophosaurins are known primarily from middle Campanian units in northern Laramidia, including the holotype of *Acristavus gagslarsoni* from the lower Two Medicine Formation of Montana (Gates et al., 2011), “*Brachylophosaurus goodwini*” and *Probrachylophosaurus bergei* from the lower Judith River Formation of Montana (Horner, 1988; Freedman Fowler & Horner, 2015), *Brachylophosaurus canadensis* from the Comrey Sandstone Zone in the Oldman Formation of Alberta and correlative middle Judith River Formation of Montana (Prieto-Márquez, 2005; Cuthbertson & Holmes, 2010), and *Maiasaura peeblesorum* from the middle Two Medicine Formation of Montana (Horner, 1983; Prieto-Márquez & Guenther, 2018) (Fig. 1). *Brachylophosaurus canadensis* and *M*. *peeblesorum* are both known from numerous individuals covering a range of ontogenetic stages. *Maiasaura* sp. indet. has also been identified from the Comrey Sandstone Zone in the Oldman Formation of southernmost Alberta (McFeeters et al., 2020).

Brachylophosaurins are comparatively sparsely known from southern Laramidia, making the occurrences of the few available specimens significant (Fig. 1). An incomplete juvenile hadrosaurid skeleton from the Mancos Shale of Colorado is the oldest known brachylophosaurin (between 81 and 80 Ma) (Lucas et al., 2006; Gates et al., 2011; Fowler, 2017). A flat-headed partial skull from the Wahweap Formation of Utah has been referred to *Acristavus gagslarsoni* (Gates et al., 2011). An isolated jugal from the Kaiparowits Formation of Utah represents the latest occurrence of Brachylophosaurini in Laramidia (~76.5 Ma) (Gates et al., 2013; Fowler, 2017). The holotype of the new brachylophosaurin from the Menefee Formation is the southernmost occurrence of the clade and the first occurrence from New Mexico.

## Materials and Methods

WSC 10058 was collected under permit NM 18-03S, issued by the U.S. Bureau of Land Management.

The electronic version of this article in Portable Document Format (PDF) will represent a published work according to the International Commission on Zoological Nomenclature (ICZN), and hence the new names contained in the electronic version are effectively published under that Code from the electronic edition alone. This published work and the nomenclatural acts it contains have been registered in ZooBank, the online registration system for the ICZN. The ZooBank LSIDs (Life Science Identifiers) can be resolved and the associated information viewed through any standard web browser by appending the LSID to the prefix http://zoobank.org/. The LSID for this publication is: urn:lsid:zoobank.org:pub:BA68A73C-5628-47FB-8EB0-3A8EC54F42EF. The online version of this work is archived and available from the following digital repositories: PeerJ, PubMed Central and CLOCKSS.

### Phylogenetic analysis

The phylogenetic analysis employed a modified version of the matrix used by McDonald et al. (2017). Several new non-hadrosaurid iguanodontians were added, along with numerous taxa to represent the diversity of hadrosaurids from North America, Asia, and Europe, including the new taxon *Ornatops incantatus*. The data matrix consisted of 86 taxa and 204 characters (Data Matrix; Character List; Specimen List).

The data matrix was analyzed in TNT 1.5 (Goloboff & Catalano, 2016). We used the method employed by Brusatte & Carr (2016) in an analysis of Tyrannosauroidea, which entailed a New Technology Search followed by tree bisection reconnection. *Camptosaurus dispar* was designated as the outgroup. The matrix was first analyzed using a New Technology Search, with the default parameters for sectorial search, ratchet, tree drift, and tree fusion; a random seed of 1; 10 replicates; and the number of times to find a minimum length tree set at 10. This search examined 686,891,312 rearrangements and recovered 66 most parsimonious trees of 692 steps, consistency index of 0.436, and retention index of 0.835. These 66 trees were then examined using the tree bisection reconnection swapping algorithm, which examined 2,440,284,496 rearrangements and recovered 11,232 most parsimonious trees. The strict consensus of these 11,232 trees was then derived in TNT.

### Digitizing WSC 10058

Digital 3-D models of the elements of WSC 10058 were created at the Western Science Center through laser scanning and photogrammetry. Scanning employed a NextEngine 3D Scanner and NextEngine AutoPositioner, in concert with the ScanStudio software. The scans were further processed in Meshmixer. Photogrammetry used a Nikon D5600 camera. The images were processed in AgiSoft PhotoScan, with further refinement of the models in Autodesk Meshmixer. The 3-D models of WSC 10058 are available on the MorphoSource (Project: WSC10058) and Sketchfab websites.

## Results

### Systematic paleontology

Dinosauria Owen, 1842, sensu Baron, Norman & Barrett, 2017

Ornithischia Seeley, 1888, sensu Sereno, McAllister & Brusatte, 2005

Ornithopoda Marsh, 1881, sensu Butler, Upchurch & Norman, 2008

Iguanodontia Baur, 1891, sensu Sereno, McAllister & Brusatte, 2005

Hadrosauridae Cope, 1869, sensu Prieto-Márquez, 2010a

Saurolophinae Brown, 1914, sensu Prieto-Márquez, 2010a

Brachylophosaurini Gates et al., 2011, sensu Freedman Fowler & Horner, 2015

*Ornatops incantatus* gen. et sp. nov.

Holotype: WSC 10058, associated skeleton including the partial right premaxilla, right postorbital, right squamosal, both quadrates, nearly complete skull roof and braincase, two partial dorsal vertebrae, a dorsal rib, ossified tendons, the right scapula, proximal end of the right humerus, right ulna lacking the proximal end, right radius lacking the proximal end, right metacarpals II and III, and incomplete pubis and ischium. While this article focuses on the cranial bones, the rest of the specimen will be completely described in a forthcoming publication (A.T. McDonald et al., 2021, in preparation).

Etymology: *Ornatops* is derived from the Latin word *ornatus* (ornate) and the Greek *ops* (face), in reference to the elaborate nasofrontal suture. The species name, *incantatus*, is a Latin word meaning “enchanted”, referring to the State of New Mexico, where the holotype was collected and which carries the motto “Land of Enchantment”. The binomen can be translated as “enchanted ornate face”.

Locality: WSC 10058 was collected in San Juan County, New Mexico, on land administered by the United States Bureau of Land Management (BLM). Precise locality data are on file at WSC and the BLM.

Horizon: WSC 10058 was collected at a single locality in the Juans Lake Beds (Miller, Carey & Thompson-Rizer, 1991), upper part of the Allison Member, Menefee Formation, approximately 120 meters below the overlying Cliff House Sandstone; middle Campanian, Upper Cretaceous, older than ~78.5 Ma based upon the occurrence of the ammonite index fossil *Baculites perplexus* in the overlying Cliff House Sandstone (Siemers & King, 1974; Molenaar et al., 2002; Lucas et al., 2005).

Specific diagnosis (as for genus by monotypy): brachylophosaurin distinguished by a single autapomorphy: nasofrontal suture on dorsal surface of frontals is horizontal rostrally and elevated caudally, ending in a pair of parasagittal bumps adjacent to the cranial midline. Furthermore, the nasofrontal suture extends caudally onto the dorsal surface of the frontals farther than in adult *Probrachylophosaurus bergei*, but not as far as in adult *Brachylophosaurus canadensis*.

**Description**

Measurements of the cranial bones of WSC 10058 are provided in Table 1. WSC 10058 was collected at a single locality and includes no duplicated or size-incompatible elements that would indicate the presence of more than one hadrosaurid individual. The only other fossils observed at the locality were fragments of crocodylomorph osteoderms situated approximately one meter stratigraphically higher than WSC 10058.

**Table 1:** Table of Cranial Measurements. Measurements of WSC 10058, holotype of *Ornatops incantatus* gen. et sp. nov.

Elements	Measurements (cm)
Right postorbital	
Preserved rostrocaudal length, from rostral-most point on frontal process to caudal-most point on squamosal process	12.2
Preserved dorsoventral height, from ventral-most point on jugal process to dorsal-most point vertically above it	6.6
Maximum mediolateral thickness of the orbital rim	4.3
Right squamosal	
Preserved rostrocaudal length, from rostral-most point on postorbital process to caudal margin	10.6
Preserved mediolateral width, from base of postquadrate process to medial tip of caudomedial process	12.2
Left quadrate	
Dorsoventral height	31.8
Dorsoventral height of quadratojugal notch	10.0
Mediolateral width of ventral end	5.1
Frontals	
Rostrocaudal length at midline	9.6
Rostrocaudal length of nasofrontal suture at midline	6.2
Preserved maximum width	10.2

**Premaxilla**

**Figure 2:**
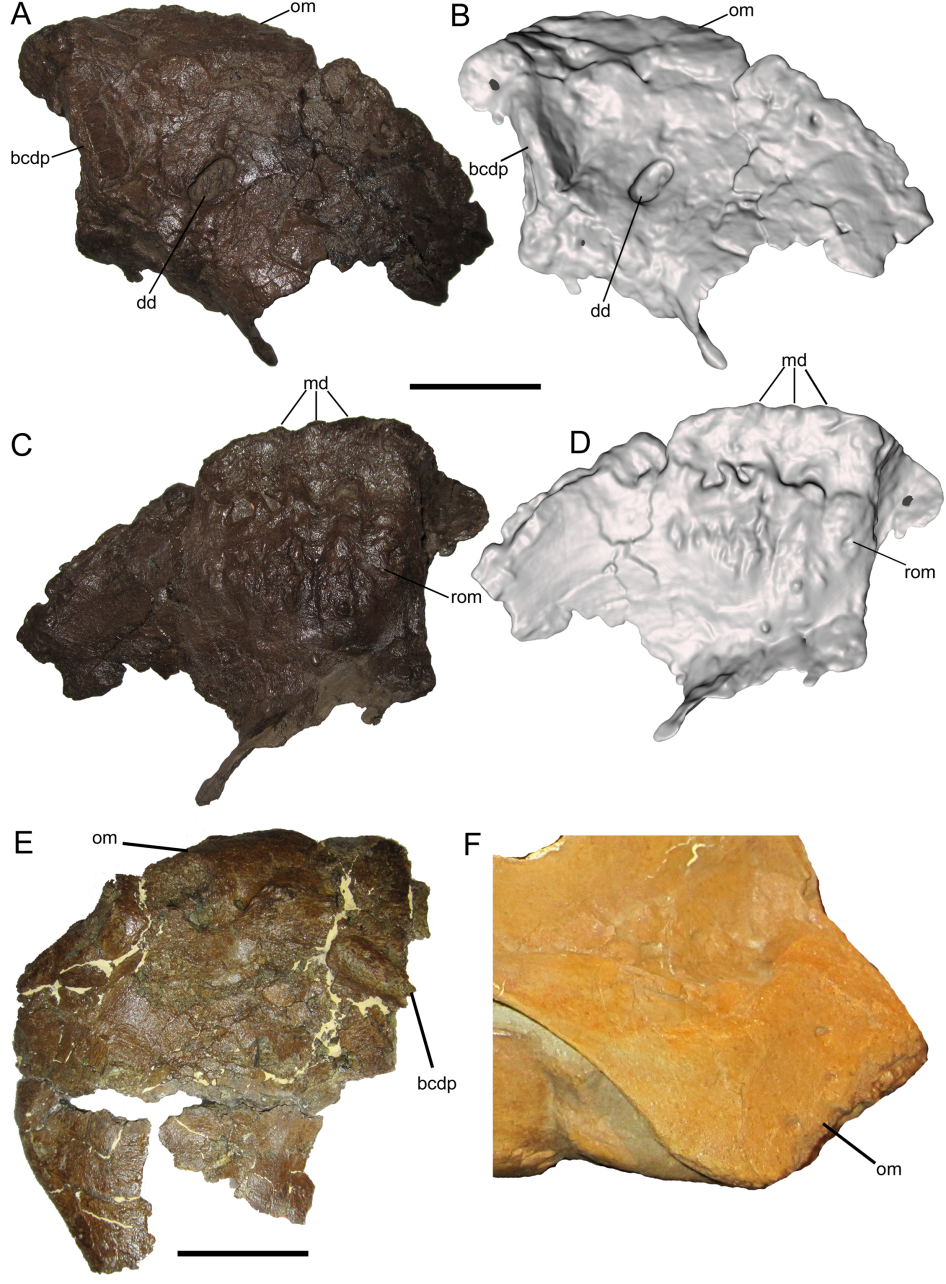
Premaxilla of WSC 10058, holotype of *Ornatops incantatus*. Right premaxilla and digital 3D model in dorsal (A and B) and ventral (C and D) views. (E) Oral portion of left premaxilla of MOR 2919 (*Probrachylophosaurus bergei*) in dorsal view. (F) Oral portion of right premaxilla of MOR 794 (*Brachylophosaurus canadensis*) in lateral view. Abbreviations: bcdp, base of caudodorsal process; dd, dorsal depression; md, marginal denticles; om, oral margin; rom, rugosity on oral margin. Scale bars in A–E equal 5 cm.

The right premaxilla is represented only by the rostrolateral portion, including the oral margin but lacking the caudodorsal and caudolateral processes (Figs. 2A–2D). The dorsal surface is weathered, but several salient features can be recognized. A portion of the broken base of the caudodorsal process is preserved adjacent to the interpremaxillary suture. Lateral to the base of the caudodorsal process is a deep, sharply defined, oblong depression, with its long axis oriented caudomedially to rostrolaterally (Figs. 2A and 2B). Similar depressions are present on premaxillae of *Probrachylophosaurus bergei* (MOR 2919) and *Brachylophosaurus canadensis* (MOR 794). Rostral and lateral to this large depression are two smaller neurovascular foramina, located closer to the lateral margin of the premaxilla. As in other brachylophosaurins (Gates et al., 2011) (Figs. 2E and 2F), the rostrodorsal surface lacks a reflected rim and instead slopes gently down to the oral margin.

The ventral surface of the premaxilla bears three small denticles along the rostral margin (Figs. 2C and 2D). Caudal to the denticles are three deep sulci that define the rostral margin of a dorsoventrally thick, ventrally convex, highly rugose area that would have supported part of the keratinous rhamphotheca that enveloped the oral region of the premaxilla in hadrosaurs (Morris, 1970; Gates & Sampson, 2007; Farke et al., 2013). The rugosities are roughly linear and extend caudally as a series of ridges, bumps, and furrows. Caudolateral to this rugose area, the ventral surface of the premaxilla becomes smooth and dorsally arched.

**Postorbital**

**Figure 3:**
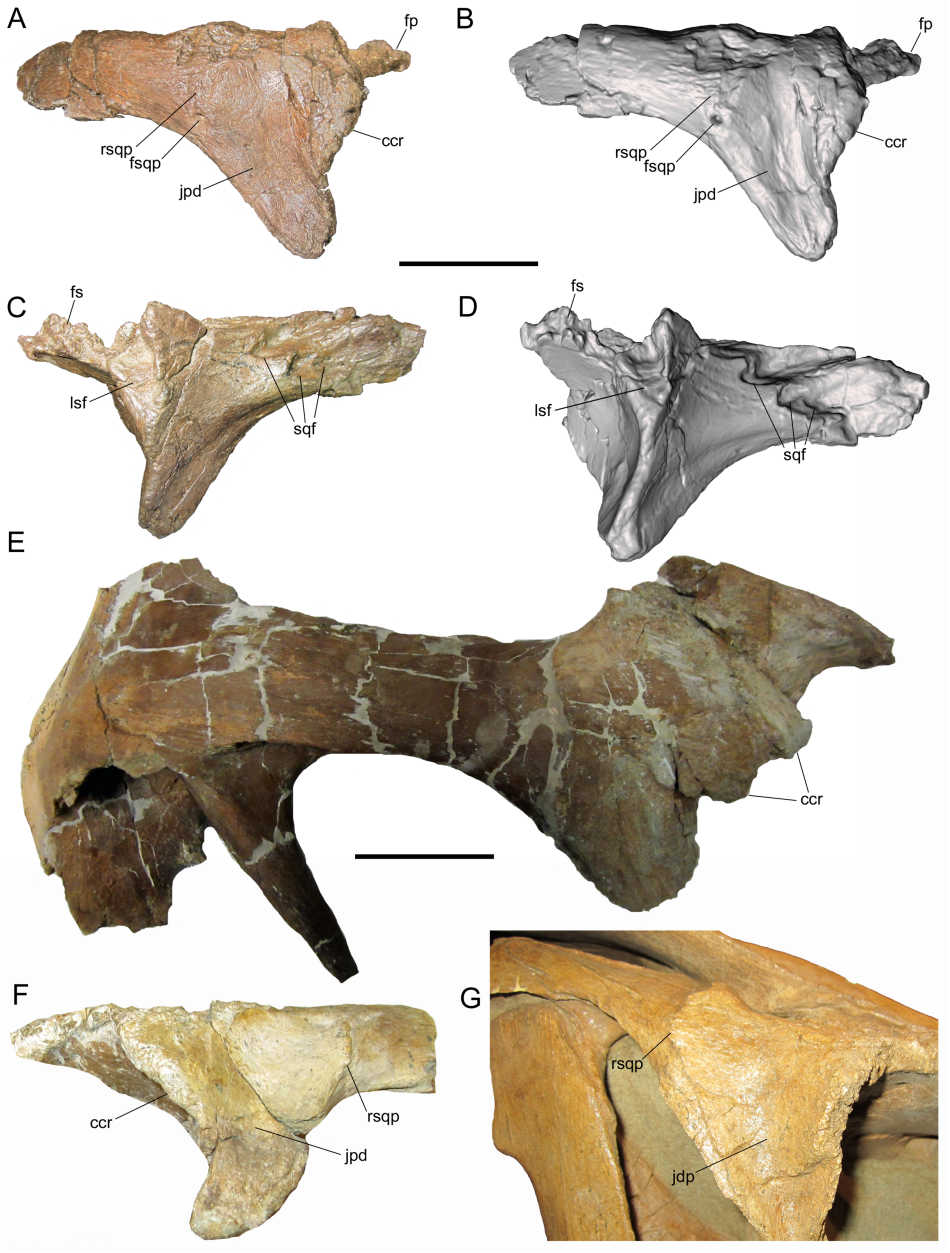
Postorbitals of WSC 10058, holotype *Ornatops incantatus*, and other brachylophosaurins. Right postorbital and digital 3D model of WSC 10058 (*Ornatops incantatus*) in lateral (A and B) and medial (C and D) views. Right postorbital and squamosal of MOR 720 (*Brachylophosaurus canadensis*) in lateral (E) view. Left postorbital of UMNH VP 16607 (*Acristavus gagslarsoni*) in lateral (F) view, with surrounding cranial bones digitally removed (Courtesy of Natural History Museum of Utah). Right postorbital and surrounding bones of MOR 794 (*Brachylophosaurus canadensis*) in lateral (G) view. Abbreviations: ccr, convex caudodorsal rim; fp, frontal process; fs, frontal suture; fsqp, foramen on squamosal process; jpd, depression on jugal process; lsf, facet for contact with laterosphenoid; rsqp, vertical ridge on squamosal process; sqf, facets for contact with squamosal. Scale bars for A–E equal 5 cm.

The right postorbital is nearly complete, missing only a portion of the frontal process, the ventral end of the jugal process, and the caudal end of the squamosal process. The frontal process extends rostrally and exhibits part of the rugose interdigitating suture with the frontal on its medial surface (Figs. 3A–3D). The concave orbital margin is defined dorsally by the frontal process, caudodorsally by the body of the postorbital, and caudoventrally by the jugal process. A mediolaterally thick rim is present on the rostrolateral margin of the body of the postorbital, projecting into the caudodorsal corner of the orbit. This rim is rostrally convex, breaking up the otherwise smoothly concave lateral orbital margin (Figs. 3A and 3B). A similar convex rim is present on the right postorbital of MOR 720, a large incomplete skull of *Brachylophosaurus canadensis* (Fig. 3E), as well as the postorbitals of the holotype (MOR 1155) and referred specimen (UMNH VP 16607) of *Acristavus gagslarsoni* (also noted by Gates et al. (2011) and Freedman Fowler & Horner (2015)) (Fig. 3F).

Caudal to the convex rim, the lateral surface of the jugal process exhibits a broad, shallow depression, as in *Acristavus gagslarsoni* (MOR 1155 and UMNH VP 16607; also noted by Gates et al. (2011) and Freedman Fowler & Horner (2015)), and some specimens of *Brachylophosaurus canadensis* (e.g., MOR 794, 1071 7-7-98-86, 1071 7-16-98-248; also noted by Freedman Fowler & Horner (2015)) (Fig. 3F and 3G). Caudal to this depression, near the ventral margin of the squamosal process, is a large neurovascular foramen that opens laterally (Figs. 3A and 3B); a similar foramen in the same position is present on the postorbital of the non-hadrosaurid hadrosauromorph *Jeyawati rugoculus* (MSM P4166) from the Turonian Moreno Hill Formation of New Mexico (McDonald, Wolfe & Kirkland, 2010). A foramen is also present on the lateral surface of the squamosal process, albeit more dorsally situated, in the basal hadrosauroids *Jinzhousaurus yangi* from the Aptian Dakangpu Member of the Yixian Formation of China (Barrett et al., 2009), and *Eolambia caroljonesa* from the Cenomanian Mussentuchit Member of the Cedar Mountain Formation of Utah (McDonald et al., 2012). This feature is variable in *Brachylophosaurus canadensis*, with the foramen absent in most adult-sized skulls (e.g., MOR 720, 794, 1071 7-7-98-86); it is present in MOR 1071 7-16-98-248, but only on the left postorbital.

Dorsal to the large neurovascular foramen, the lateral surface of the postorbital is damaged. However, the lateral surface immediately dorsal to the foramen is strongly and sharply convex, indicating the presence of a vertical ridge at the base of the squamosal process (Figs. 3A and 3B); the dorsal end of this ridge is preserved near the dorsal margin of the squamosal process. The presence of the vertical ridge is variable in *Acristavus gagslarsoni* (absent in MOR 1155; present in UMNH VP 16607 (also noted by Gates et al. (2011))) and *Brachylophosaurus canadensis* (absent in MOR 720, 1071 7-7-98-86, and 1071 7-16-98-248; present in MOR 794) (Figs. 3E–3G).

The medial surface of the postorbital is dominated by a series of contact surfaces for adjacent bones, including the aforementioned rugose frontal suture. Caudal to the frontal suture is a facet to receive the postorbital process of the laterosphenoid (Figs. 3C and 3D). This facet appears more oblong dorsoventrally than the circular laterosphenoid facets of other brachylophosaurin specimens (e.g., MOR 720 (*Brachylophosaurus*), MOR 2919 (*Probrachylophosaurus*)), but this shape is perhaps somewhat exaggerated due to damage to the facet’s dorsal margin and slight rostral displacement of the section of bone that forms its caudal margin. The medial surface of the squamosal process of the postorbital bears three rostrocaudally-elongate facets with which the postorbital process of the squamosal would have articulated (Figs. 3C and 3D). The dorsal and rostral-most facet is the deepest. Caudoventral to this is a second, similarly pronounced facet. Caudal to this second facet is a third, shallower facet. A gentle ridge that fits into this third, shallower facet is preserved on the lateral surface of the postorbital process of the squamosal (see below).

**Squamosal**

**Figure 4:**
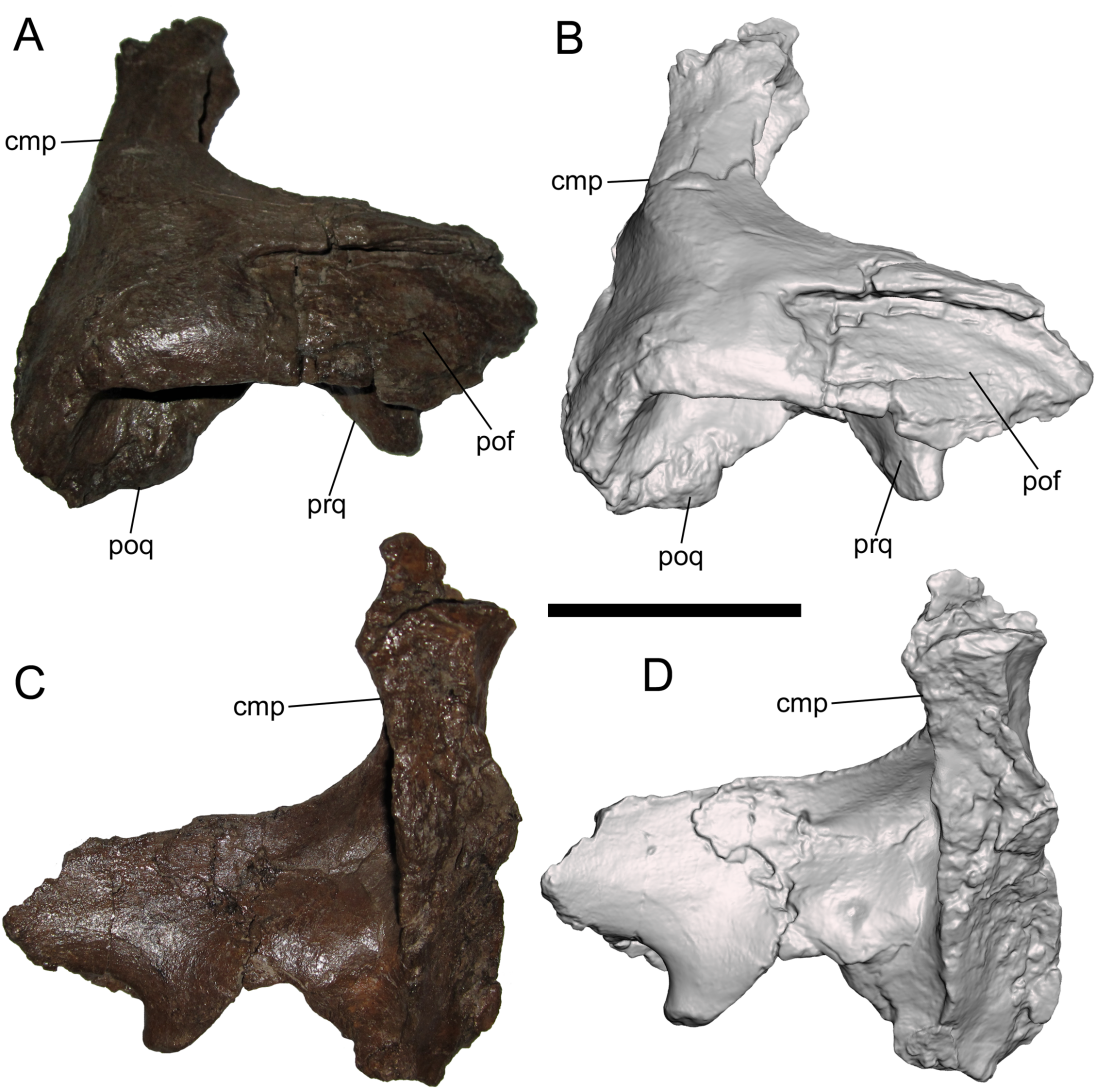
Squamosal of WSC 10058, holotype of *Ornatops incantatus*. Right squamosal and digital 3D model in lateral (A and B) and medial (C and D) views. Abbreviations: cmp, caudomedial process; pof, facet for contact with postorbital; poq, postquadrate process; prq, prequadrate process. Scale bar equals 5 cm.

The right squamosal is nearly complete except for the rostral end of the postorbital process, and the prequadrate and postquadrate processes, of which only the bases are preserved. The lateral surface of the postorbital process exhibits a well-defined facet to receive the squamosal process of the postorbital (Figs. 4A and 4B). The postorbital and squamosal of WSC 10058 no longer articulate tightly due to damage to the rostral end of the postorbital process of the squamosal where it would have fit into the aforementioned two more prominent facets on the medial surface of the squamosal process of the postorbital (see above). However, the articulation facet on the postorbital process of the squamosal bears a slight rostrocaudally-oriented ridge that fits into the aforementioned third, shallower facet on the medial surface of the squamosal process of the postorbital (see above). The caudal end of the articulation facet on the postorbital process is shallowly bifurcated, indicating that the caudal end of the squamosal process of the postorbital was also bifurcated.

The caudomedial process is straight and projects medially (Figs. 4C and 4D). The rostral aspect of the caudomedial process forms a deep recess bounded by two thin laminae, providing an origin site for the M. adductor mandibulae externus medialis at the caudal margin of the supratemporal fenestra, as in other hadrosaurids (Ostrom, 1961). The medial surface of the caudomedial process exhibits a dorsoventrally-elongate shallow facet, forming the contact surface with the exoccipital-opisthotic.

**Quadrates**

**Figure 5:**
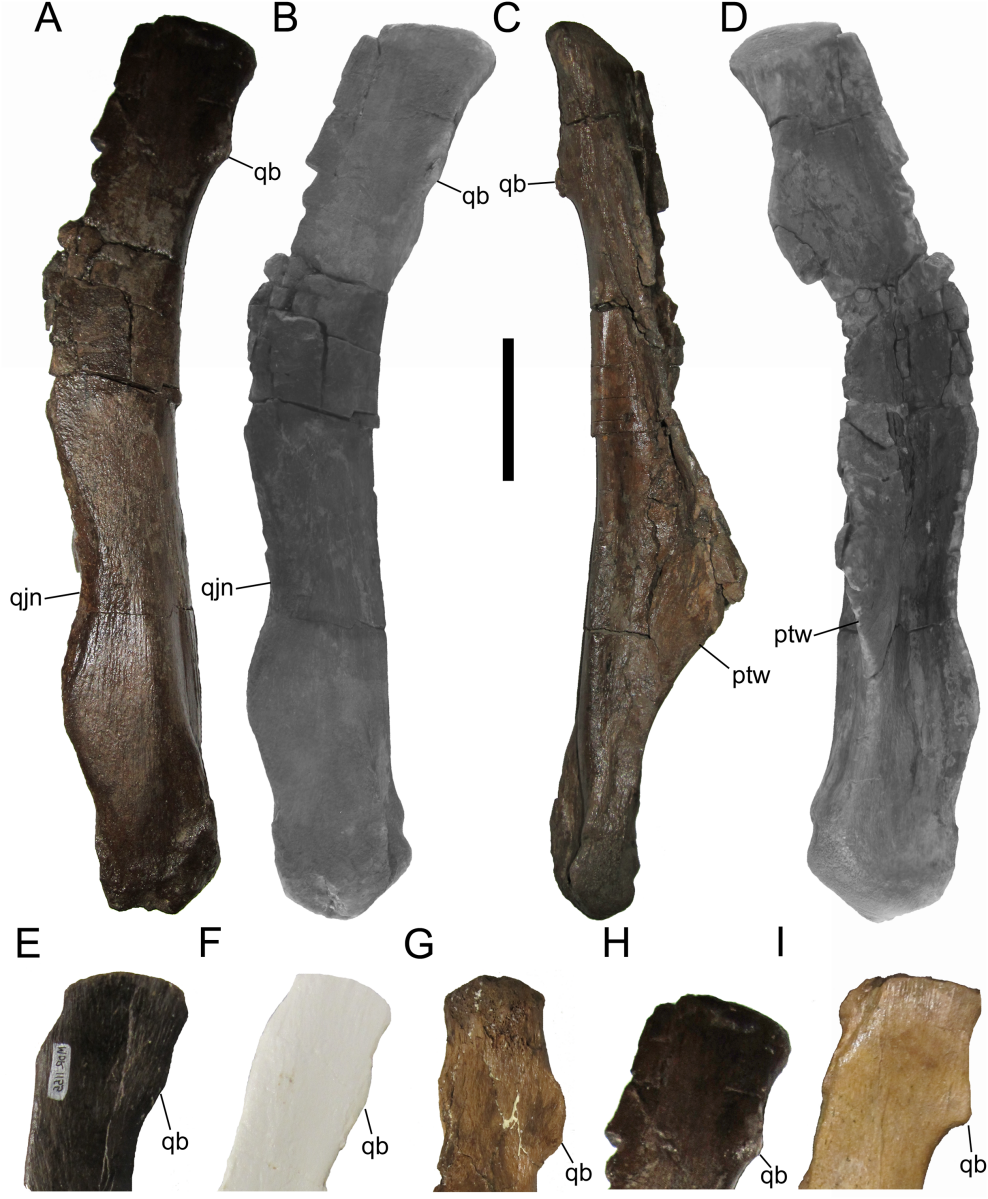
Quadrates of WSC 10058, holotype of *Ornatops incantatus*, and other brachylophosaurins. Left quadrate and digital 3D model of WSC 10058 (*Ornatops incantatus*) in lateral (A and B), medial (C), and rostral (D) views. Dorsal ends of brachylophosaurin quadrates: (E) *Acristavus gagslarsoni* MOR 1155 right quadrate in lateral view (reversed); (F) *Maiasaura peeblesorum* MOR CAST 089 (replica of TMDC/OTM F138) right quadrate in lateral view (reversed); (G) *Probrachylophosaurus bergei* MOR 2919 right quadrate in medial view; (H) *Ornatops incantatus* WSC 10058 left quadrate in lateral view; and (I) *Brachylophosaurus canadensis* MOR 794 right quadrate in lateral view (reversed), with surrounding cranial bones digitally removed. Abbreviations: ptw, pterygoid wing; qb, quadrate buttress; qjn, quadratojugal notch. Scale bar equals 5 cm for A–D.

WSC 10058 includes both quadrates, but the right quadrate is heavily weathered and missing the dorsal and ventral condyles, so this description focuses on the nearly complete left quadrate. The left quadrate measures 31.8 cm tall, 98% the size of the quadrates of MOR 794 (32.5 cm), an adult specimen of *Brachylophosaurus canadensis* (Freedman Fowler & Horner, 2015). The quadrate curves gently caudally along its entire dorsoventral height (Figs. 5A–5D). The ventral condyle is mediolaterally broad, while the dorsal condyle is mediolaterally compressed and rostrocaudally elongate. The quadratojugal notch in the lateral wing of the quadrate is dorsoventrally broad and shallow, with an elongate facet along its rostrolateral margin for articulation with the quadratojugal (Figs. 5A and 5B). The pterygoid wing projects rostromedially and exhibits a deep recess on its medial surface (Figs. 5C and 5D). On the caudolateral margin of the quadrate shaft, immediately ventral to the dorsal condyle, is a distinct and prominent quadrate buttress, similar to the pronounced, angular buttresses in *Probrachylophosaurus bergei* (MOR 2919) (Freedman Fowler & Horner, 2015) and *Brachylophosaurus canadensis* (e.g., MOR 794 and 1071 8-13-98-589-D) (Prieto-Márquez, 2005; Cuthbertson & Holmes, 2010; Freedman Fowler & Horner, 2015), rather than the low, rounded buttresses in *Acristavus gagslarsoni* (MOR 1155) (Gates et al., 2011) and *Maiasaura peeblesorum* (e.g., MOR CAST 089 replica of TMDC/OTM F138) (Horner, 1983; Trexler, 1995) (Figs. 5E–5I).

**Braincase**

**Figure 6:**
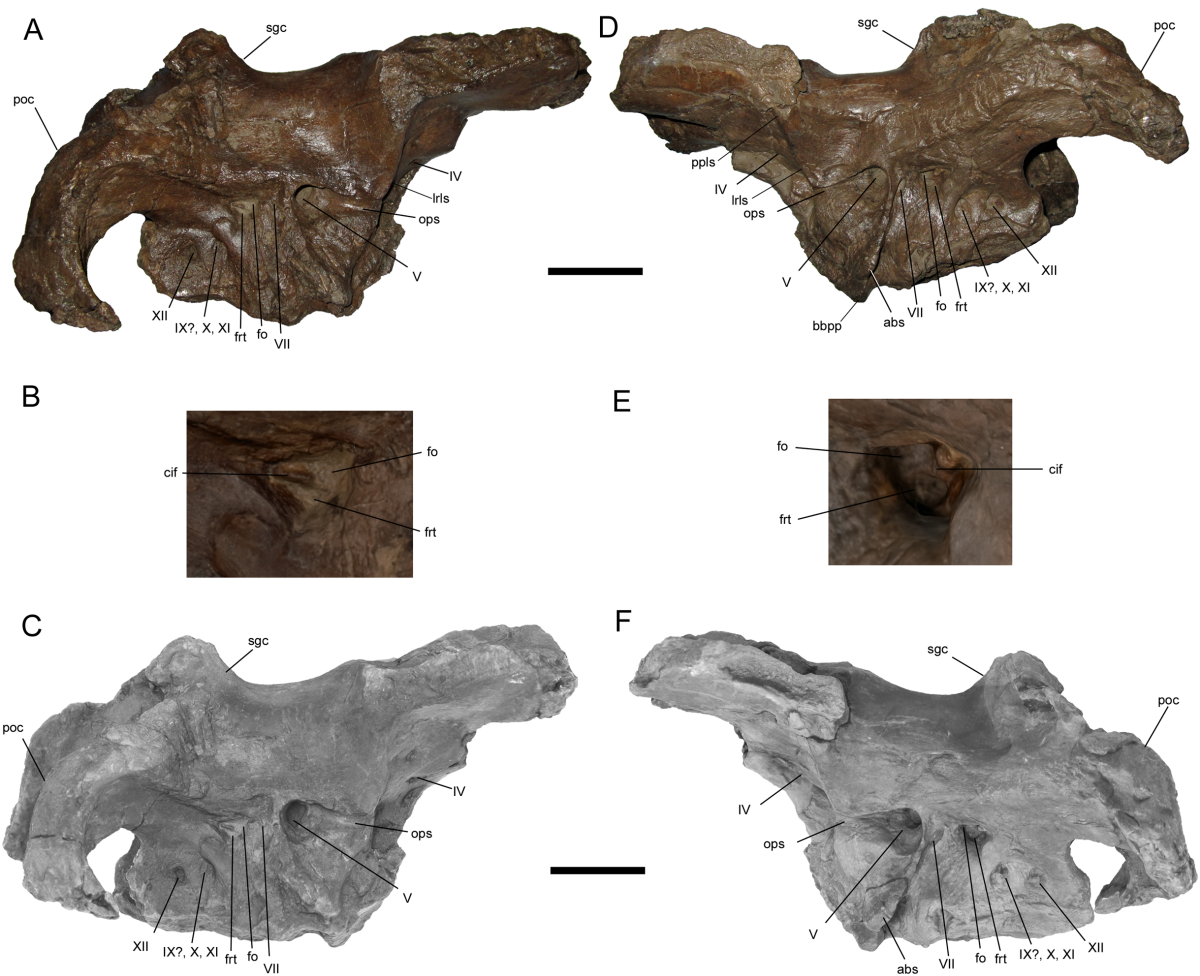
Braincase of WSC 10058, holotype of *Ornatops incantatus*. Braincase and digital 3D model in right lateral (A and C) and left lateral (D and F) views, with enlarged images of the right (B) and left (E) auditory regions. Abbreviations: abs, alar process of the basisphenoid; bbpp, base of basipterygoid process; cif, crista interfenestralis; fo, fenestra ovalis; frt, fenestra rotunda; lrls, lateral ridge on laterosphenoid; ops, ophthalmic sulcus; poc, paroccipital process; ppls, postorbital process of laterosphenoid; sgc, sagittal crest; IV–XII, cranial nerve exits. Scale bars in A, C, D, F equal 5 cm.

**Figure 7:**
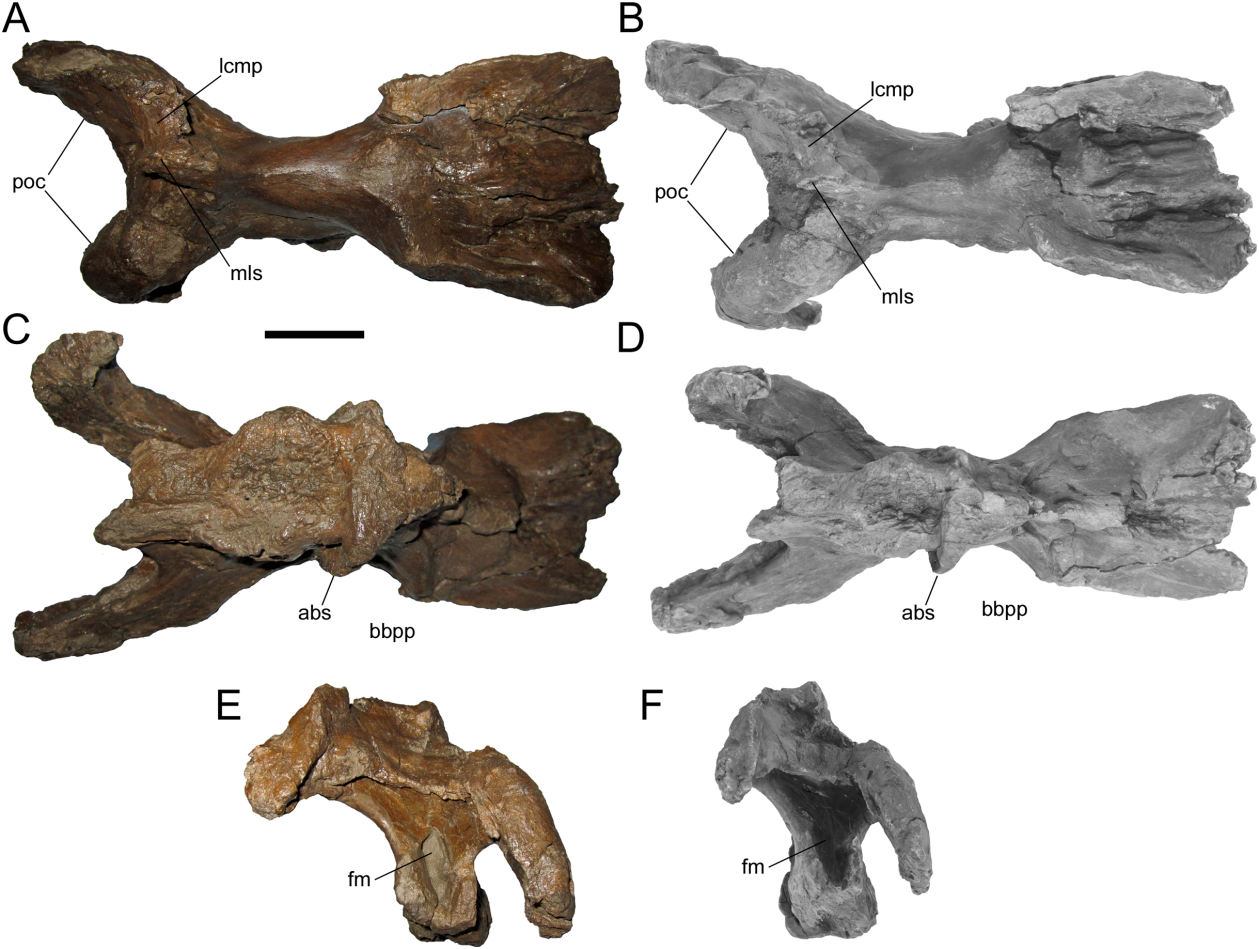
Braincase of WSC 10058, holotype of *Ornatops incantatus*. Braincase and digital 3D model in dorsal (A and B), ventral (C and D), and caudal (E and F) views. Abbreviations: abs, alar process of the basisphenoid; bbpp, base of basipterygoid process; fm, foramen magnum; lcmp, partial caudomedial process of left squamosal; mls, midline suture between caudomedial processes of the squamosals; poc, paroccipital process. Scale bar equals 5 cm.

The braincase of WSC 10058 is nearly complete, apart from damage to the basicranium, such as breakage of the occipital condyle and abrasion of the ventral surfaces of the basisphenoid and basioccipital; the broken base of the left basipterygoid process is preserved on the ventral surface of the basisphenoid. The braincase is also plastically deformed along the sagittal plane, with the entire right side shifted ventrally relative to the left (Figs. 6 and 7). As in the adult holotypes of *Probrachylophosaurus bergei* (Freedman Fowler & Horner, 2015) and *Brachylophosaurus canadensis* (Cuthbertson & Holmes, 2010), the sutures between the bones of the braincase are fused and remodeled, suggesting a late ontogenetic stage. However, despite the damage, deformation, and sutural co-ossification, much of the detailed anatomy, particularly the cranial nerve openings, is well-preserved.

The postorbital process of the left laterosphenoid is preserved, displaying the blunt articulation surface that would contact the aforementioned facet on the medial surface of the postorbital (Figs. 6D and 6F). A prominent lateral ridge extends ventrally from the postorbital process until it meets the rostral end of another, rostrocaudally-oriented ridge that defines the overhanging dorsal margin of the ophthalmic sulcus.

A large portion of the left alar process of the basisphenoid is preserved. It is laterally-directed and dorsoventrally deep, with a base that extends ventrally from a point immediately caudoventral to the trigeminal nerve opening to a point on the base of the basipterygoid process (Figs. 6D, 6F, 7C and 7D). The alar process completely shrouds the foramen for the internal carotid artery (Ostrom, 1961). The dorsoventral extent of the alar process of WSC 10058 is comparable to those of other brachylophosaurins (e.g., *Acristavus* (MOR 1155, UMNH VP 16607), *Probrachylophosaurus* (MOR 2919), *Brachylophosaurus* (MOR 1071 7-7-98-86, MOR 1071 7-16-98-248)) (Prieto-Márquez, 2005; Cuthbertson & Holmes, 2010; Gates et al., 2011; Freedman Fowler & Horner, 2015). An enlarged alar process is a diagnostic character of Brachylophosaurini (Gates et al., 2011). This morphology differs from the dorsoventrally shallower alar processes of other saurolophines, such as *Gryposaurus notabilis* (Prieto-Márquez, 2010b), *Saurolophus angustirostris* (Bell, 2011a), and *Edmontosaurus regalis* (Xing, Mallon & Currie, 2017).

Most of the cranial nerve exits are well-preserved on both sides of the braincase (Fig. 6), and have been identified based upon prior descriptions of brachylophosaurin braincases (Prieto-Márquez, 2005; Cuthbertson & Holmes, 2010; Gates et al., 2011; Freedman Fowler & Horner, 2015), other saurolophine braincases (Bolotsky & Godefroit, 2004; Prieto-Márquez, 2010b; Bell, 2011a, 2011b; Godefroit, Bolotsky & Lauters, 2012; McGarrity, Campione & Evans, 2013; Xing, Mallon & Currie, 2017), and the work of Ostrom (1961). Crushing of the presphenoid and parasphenoid process has obscured the regions of the exits for c.n.I (olfactory nerve), c.n.II (optic nerve), and the common exit for c.n.III (oculomotor nerve) and c.n.VI (abducens nerve). The small, rostrocaudally-elongate foramen for c.n.IV (trochlear nerve) is near the caudoventral margin of the orbitosphenoid. This is similar to some other saurolophines, in which a single trochlear foramen pierces the body of the orbitosphenoid, including *Gryposaurus notabilis* (Prieto-Márquez, 2010b), *Kerberosaurus manakini* (Bolotsky & Godefroit, 2004), and *Edmontosaurus regalis* (Xing, Mallon & Currie, 2017). Prieto-Márquez (2005) identified two trochlear foramina in *Brachylophosaurus canadensis*, an elongated ventral foramen in the orbitosphenoid and a dorsal foramen in the laterosphenoid near the caudal margin of the orbitosphenoid. WSC 10058 does not appear to have a dorsal trochlear foramen.

The exit for c.n.V (trigeminal nerve) is the largest and opens rostrally into a deep horizontal ophthalmic sulcus, as in *Acristavus gagslarsoni* (MOR 1155, UMNH VP 16607) (Gates et al., 2011), *P*. *bergei* (MOR 2919) (Freedman Fowler & Horner, 2015), and *B*. *canadensis* (MOR 1071 7-7-98-86, 1071 7-16-98-248) (Prieto-Márquez, 2005; Cuthbertson & Holmes, 2010). The exit for c.n.VII (facial nerve) is located caudoventral to c.n.V, as in *P*. *bergei* and *B*. *canadensis*, rather than caudal as in *A*. *gagslarsoni* (Freedman Fowler & Horner, 2015). The exits for c.n.V and c.n.VII are separated by a ridge that extends rostroventrally and expands laterally to form the alar process of the basisphenoid.

The region associated with c.n.VIII (vestibulocochlear nerve) is directly caudal to c.n.VII and nearly as large as c.n.V, as in *P*. *bergei* (MOR 2919) (Freedman Fowler & Horner, 2015) and *B*. *canadensis* (MOR 1071 7-7-98-86, 1071 7-16-98-248) (Prieto-Márquez, 2005; Cuthbertson & Holmes, 2010). As identified by Ostrom (1961), this auditory region is divided into a rostrodorsal opening, the fenestra ovalis, and a caudoventral opening, the fenestra rotunda, separated by the crista interfenestralis (Figs. 6B and 6E). There is some uncertainty surrounding which opening is the exit for c.n.IX (glossopharyngeal nerve) in hadrosaurids. The landmark study of hadrosaurid cranial anatomy by Ostrom (1961), and descriptions of *Brachylophosaurus canadensis* (Prieto-Márquez, 2005; Cuthbertson & Holmes, 2010), *Probrachylophosaurus bergei* (Freedman Fowler & Horner, 2015), *Gryposaurus notabilis* (Prieto-Márquez, 2010b), *Saurolophus angustirostris* (Bell, 2011a), *Saurolophus osborni* (Bell, 2011b), and *Kerberosaurus manakini* (Bolotsky & Godefroit, 2004), place c.n.IX in the same opening as c.n.X (vagus nerve). However, a recent description of *Edmontosaurus regalis* by Xing, Mallon & Currie (2017) raised the possibility that c.n.IX exited through what would be the fenestra rotunda (identified by them as the “posteroventral part” of the fenestra ovalis (p. 23)). The braincase of WSC 10058 has been CT-scanned, and a digital cranial endocast will be described in a forthcoming publication (A.T. McDonald et al., 2021, in preparation), which might clarify the exit for c.n.IX in *Ornatops incantatus*. The common exit for c.n.X, c.n.XI (accessory nerve), and possibly c.n.IX is caudoventral to the fenestra rotunda, with the smaller exit for c.n.XII (hypoglossal nerve) directly caudal to it.

WSC 10058 is damaged in the same manner as MOR 2919, the holotype of *Probrachylophosaurus*, in which the caudomedial process of the right squamosal broke away very near the midline suture, while a portion of the caudomedial process of the left squamosal is still attached to the right caudomedial process and the parietal (Freedman Fowler & Horner, 2015) (Figs. 7A and 7B). A remnant of the midline suture between the caudomedial processes might be discernable on WSC 10058 (Figs. 7A and 7B). The caudal region of the skull roof of WSC 10058 exhibits the same condition as MOR 1155, the holotype of *Acristavus*, and MOR 2919, the holotype of *Probrachylophosaurus*, in which the caudomedial processes of the squamosals contact each other along the midline and co-ossify, excluding the sagittal crest of the parietal from dorsal view (Freedman Fowler & Horner, 2015). In contrast, the caudomedial processes are separated by the sagittal crest in *Brachylophosaurus* (Prieto-Márquez, 2005; Cuthbertson & Holmes, 2010; Freedman Fowler & Horner, 2015). The paroccipital processes flare caudolaterally and curve rostroventrally (Figs. 6 and 7).

**Frontals**

**Figure 8:**
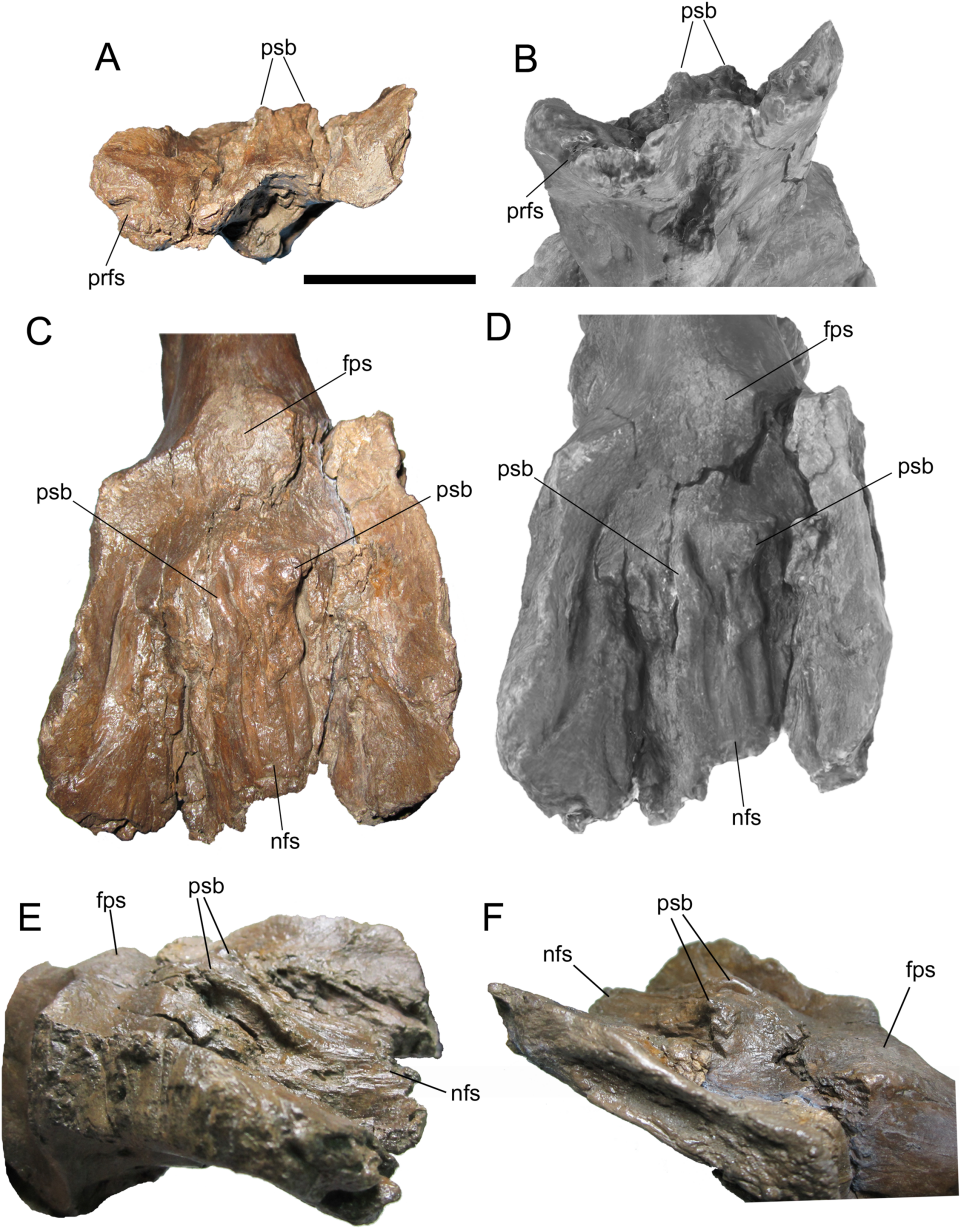
Frontals of WSC 10058, holotype of *Ornatops incantatus*. Frontals and digital 3D model in rostral (A and B) and dorsal (C and D) views. Frontals in right rostral oblique dorsolateral (E) and left caudal oblique dorsolateral (F) views. Abbreviations: fps, swelling at frontoparietal suture; nfs, nasofrontal suture; prfs, caudal end of prefrontal suture; psb, parasagittal bump at caudal end of nasofrontal suture. Scale bar equals 5 cm.

Given their importance to the diagnosis of *Ornatops incantatus*, the frontals are described separately from the rest of the braincase. Like the rest of the braincase, the frontals have suffered a degree of plastic deformation, with the lateral margins of both frontals curled dorsally and the right frontal shifted ventrally relative to the left, except along the midline of the skull (Fig. 8). Both frontals are complete except for abrasion of both postorbital sutures and breakage of both nasal processes. The caudal end of the prefrontal suture is preserved on the right frontal (Figs. 8A and 8B). The rostral margins of both frontals are intact at and adjacent to the midline of the skull, and are quite thin. The frontals gradually become dorsoventrally thicker caudally, as in *Probrachylophosaurus bergei* (MOR 2919) and *Brachylophosaurus canadensis* (MOR 1071 6-30-98-4, 1071 7-13-99-87-I, and 1071 C.3.3).

**Figure 9:**
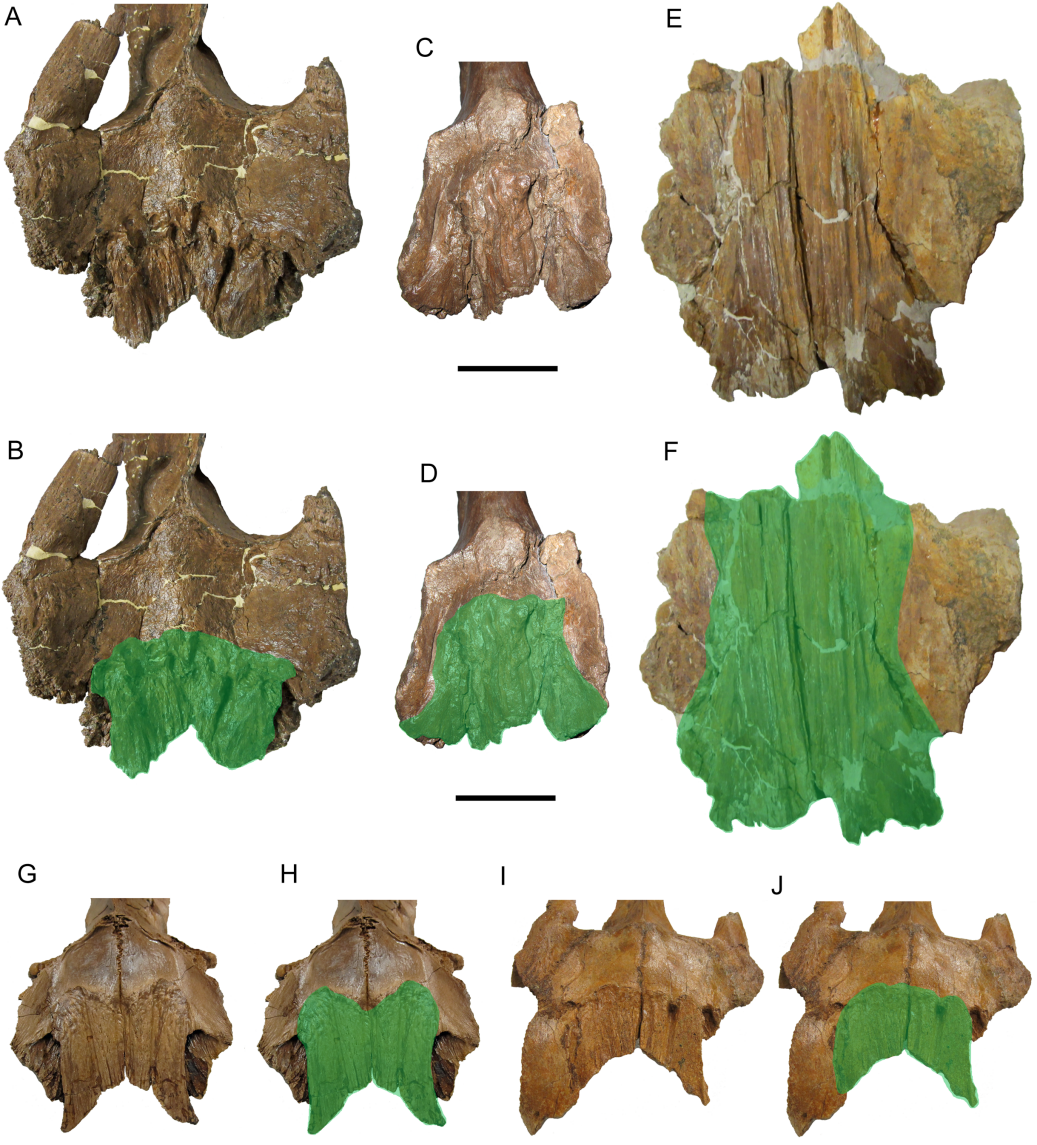
Brachylophosaurin frontals. Frontals of *Probrachylophosaurus bergei* MOR 2919 (A and B); *Ornatops incantatus* WSC 10058 (C and D); and *Brachylophosaurus canadensis* MOR 720 (E and F), MOR 1071 7-13-99-87-I (G and H), and MOR 1071 C.3.3 (I and J) in dorsal view. In B, D, F, H, and J the nasofrontal sutures are highlighted in green. Scale bars equal 5 cm.

The dorsal surface of the frontals is largely occupied by the caudally expanded nasofrontal suture (Figs. 8C–8F). This suture consists of a complex topography of rostrocaudally oriented prominent ridges and deep furrows and pockets, as in *Probrachylophosaurus bergei* (MOR 2919) (Freedman Fowler & Horner, 2015) and *Brachylophosaurus canadensis* (e.g., MOR 720, 1071 6-30-98-4, 1071 7-13-99-87-I, and 1071 C.3.3) (Prieto-Márquez, 2005; Freedman Fowler & Horner, 2015) (Figs. 8 and 9). However, in WSC 10058, the nasofrontal suture is horizontal near its rostral margin but rises caudally and ends in a pair of pronounced parasagittal bumps (Fig. 8), in contrast to the rostrocaudally horizontal nasofrontal sutures of *P*. *bergei* and *B*. *canadensis*. This caudally elevated nasofrontal suture morphology has not been observed on the frontals of the adult holotype of *P*. *bergei* (MOR 2919), nor on immature (MOR 1071 6-30-98-4, 1071 7-13-99-87-I, 1071 C.3.3) or adult (MOR 720) frontals of *B*. *canadensis*, and is an autapomorphy of *Ornatops incantatus*.

**Figure 10:**
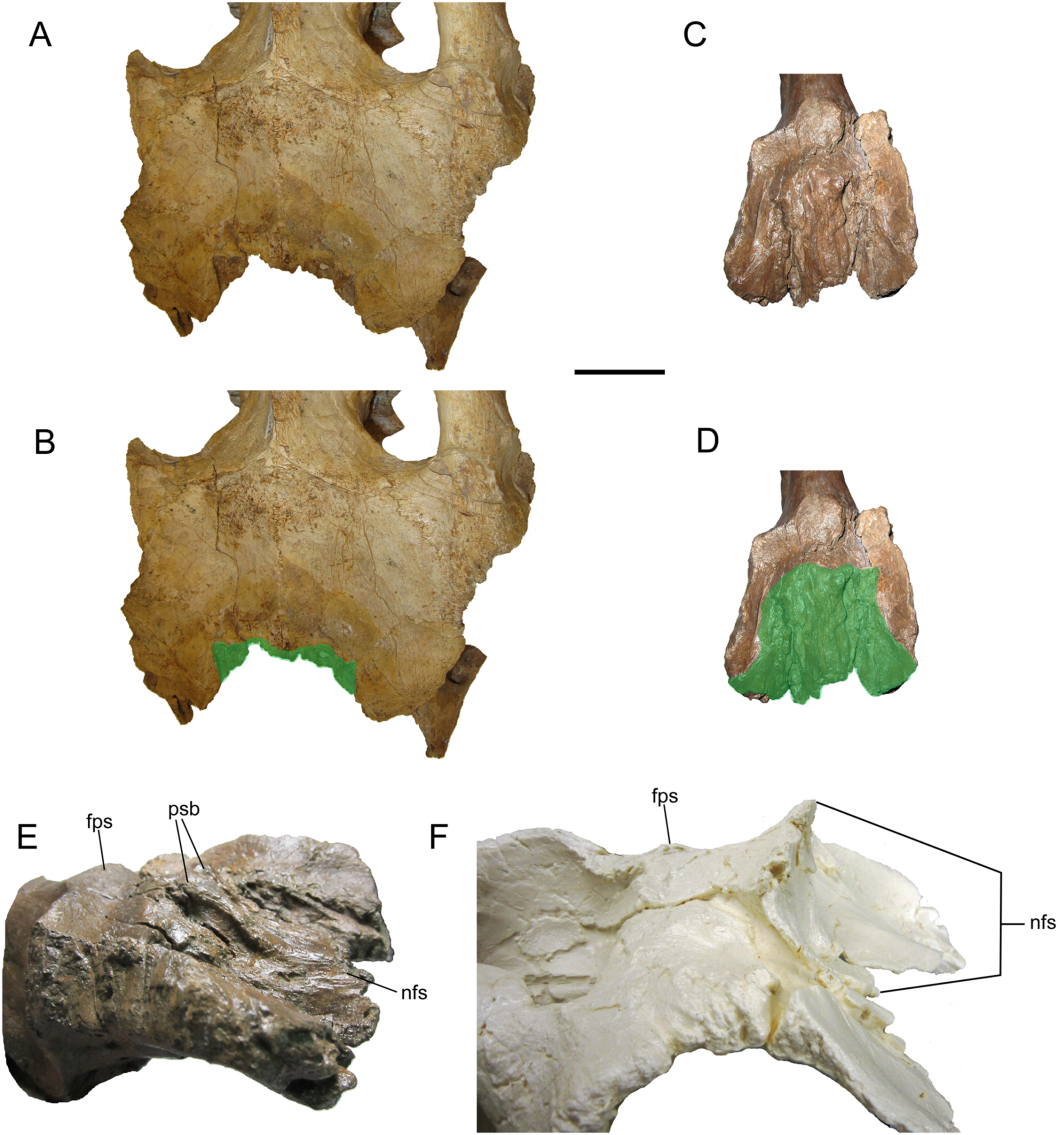
Brachylophosaurin frontals. Frontals of *Acristavus gagslarsoni* UMNH VP 16607 (A and B (Courtesy of Natural History Museum of Utah)), and *Ornatops incantatus* WSC 10058 (C and D) in dorsal view. In B and D the nasofrontal sutures are highlighted in green. Frontals of *Ornatops incantatus* WSC 10058 (E), and *Maiasaura peeblesorum* MOR CAST 089 (replica of TMDC/OTM F138) (F) in right rostral oblique dorsolateral view. Abbreviations: fps, swelling at frontoparietal suture; nfs, nasofrontal suture; psb, parasagittal bump at caudal end of nasofrontal suture. Scale bar equals 5 cm for A–D.

The nasofrontal suture of WSC 10058 also differs from that of *Acristavus gagslarsoni*, which is not caudally expanded onto the dorsal surface of the frontals and is restricted to the rostral margin (MOR 1155, UMNH VP 16607) (Gates et al., 2011) (Figs. 10A–10D). Furthermore, WSC 10058 lacks the autapomorphic squared-off caudolateral corners of the nasofrontal suture of *Acristavus* (UMNH VP 16607) (Gates et al., 2011). WSC 10058 also differs from *Maiasaura peeblesorum*, in which the nasofrontal suture is dorsoventrally deep at the rostral margin of the frontals and forms a relatively smooth, concave structure that rises vertically and curves rostrally (MOR CAST 089 replica of TMDC/OTM F138) (Horner, 1983; Trexler, 1995) (Figs. 10E and 10F).

Caudal to the nasofrontal suture, the dorsal surface of the frontals is smooth and slopes caudoventrally, before rising again at the frontoparietal suture (Figs. 8C–8F). Similar dorsal swellings at the frontoparietal suture are present in subadult *B*. *canadensis* (MOR 1071 7-13-99-87-I, 1071 C.3.3) (Prieto-Márquez, 2005) and in adult *P*. *bergei* (MOR 2919) and *Maiasaura peeblesorum* (MOR CAST 089 replica of TMDC/OTM F138) (Horner, 1983; Trexler, 1995), in contrast to the dorsally flat frontals of *Acristavus gagslarsoni* (MOR 1155, UMNH VP 16607) (Gates et al., 2011).

The extent of the nasofrontal suture on the dorsal surface of the frontals of WSC 10058 is intermediate between those of adult *Probrachylophosaurus bergei* and adult *Brachylophosaurus canadensis* (Fig. 9). In WSC 10058, the nasofrontal suture extends approximately 65% the length of the frontals (rostrocaudal length of frontals at midline = 9.6 cm, rostrocaudal length of nasofrontal suture at midline = 6.2 cm), and this is probably an underestimate, given the breakage of the left and right nasal processes. This is still greater coverage than in MOR 2919, the adult holotype of *P*. *bergei* (59%), and greater than or comparable to subadult specimens of *B*. *canadensis* (54–69%) (Freedman Fowler & Horner, 2015). In adult *B*. *canadensis*, the nasofrontal suture extends over the entire dorsal surface of the frontals (e.g., MOR 720) (Prieto-Márquez, 2005; Freedman Fowler & Horner, 2015). The extent of the nasofrontal suture in WSC 10058 is probably not attributable to immaturity; the aforementioned co-ossification of the braincase plus a quadrate similar in size to those of an adult specimen of *B*. *canadensis* suggest a late ontogenetic stage for WSC 10058. The osteohistology of WSC 10058 will be described in a future article (A.T. McDonald et al., 2021, in preparation), hopefully providing more precision as to the ontogenetic stage of the specimen.

## Discussion

**Figure 11:**
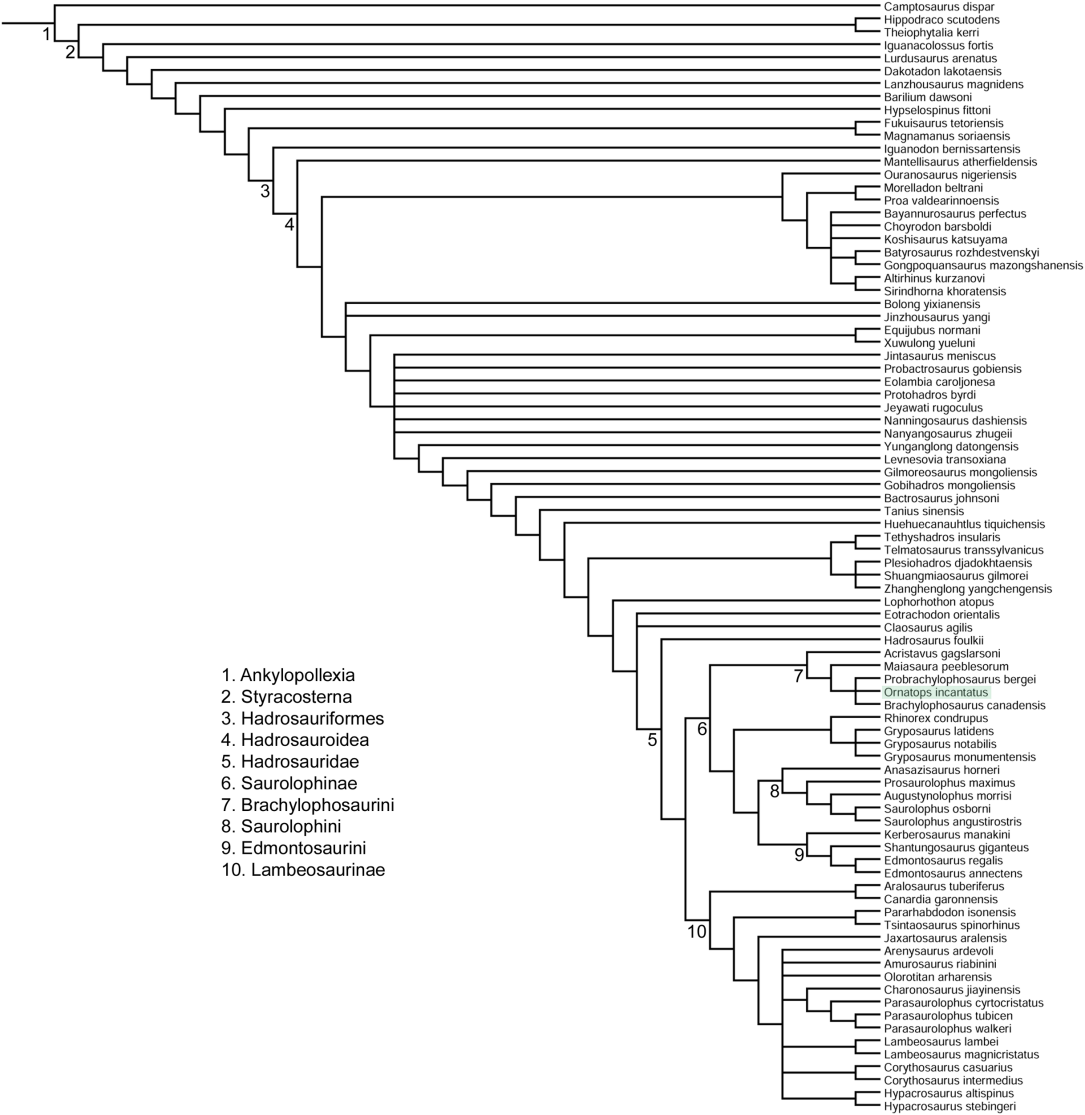
Phylogenetic relationships of *Ornatops incantatus*. Strict consensus cladogram of 11,232 most parsimonious trees obtained by TNT. Tree image was prepared in Mesquite. Major clades are numbered 1–10. *Ornatops incantatus* is highlighted in green.

The phylogenetic analysis placed *Ornatops incantatus* in a trichotomy with *Probrachylophosaurus bergei* and *Brachylophosaurus canadensis*, with *Maiasaura peeblesorum* and *Acristavus gagslarsoni* as successively more distant outgroups (Fig. 11). This result agrees with the second analysis presented by Freedman Fowler & Horner (2015, in Fig. 23), which was derived from the matrix of Gates et al. (2011), in which *P*. *bergei* and *B*. *canadensis* formed a clade to the exclusion of *M*. *peeblesorum* and *A*. *gagslarsoni*. In the current analysis, *Ornatops* shares two features with all other brachylophosaurins: lack of a dorsally everted rim along the oral margin of the premaxilla (Gates et al., 2011; Freedman Fowler & Horner, 2015), and a large alar process of the basisphenoid (Gates et al., 2011). *Ornatops* cannot currently be assessed for additional synapomorphies of Brachylophosaurini pertaining to the maxilla and jugal (71(1), 102(2), 103(2), 104(1), 106(3)) (Gates et al., 2011). The clade of (*Probrachylophosaurus* + *Ornatops* + *Brachylophosaurus*) is united by three synapomorphies, of which *Ornatops* can be coded for two: 113(1), quadrate buttress is a prominent flange well set off from shaft of quadrate and dorsal condyle; and 121(1), nasofrontal suture is transversely-wide corrugated structure that extends caudally to cover more than half of the dorsal surface of the frontals in adults. The third character is currently unknown in *Ornatops*: 66(1), transversely-wide solid nasal crest paddle-shaped, extending caudally to overhang the parietal in adults.

**Figure 12:**
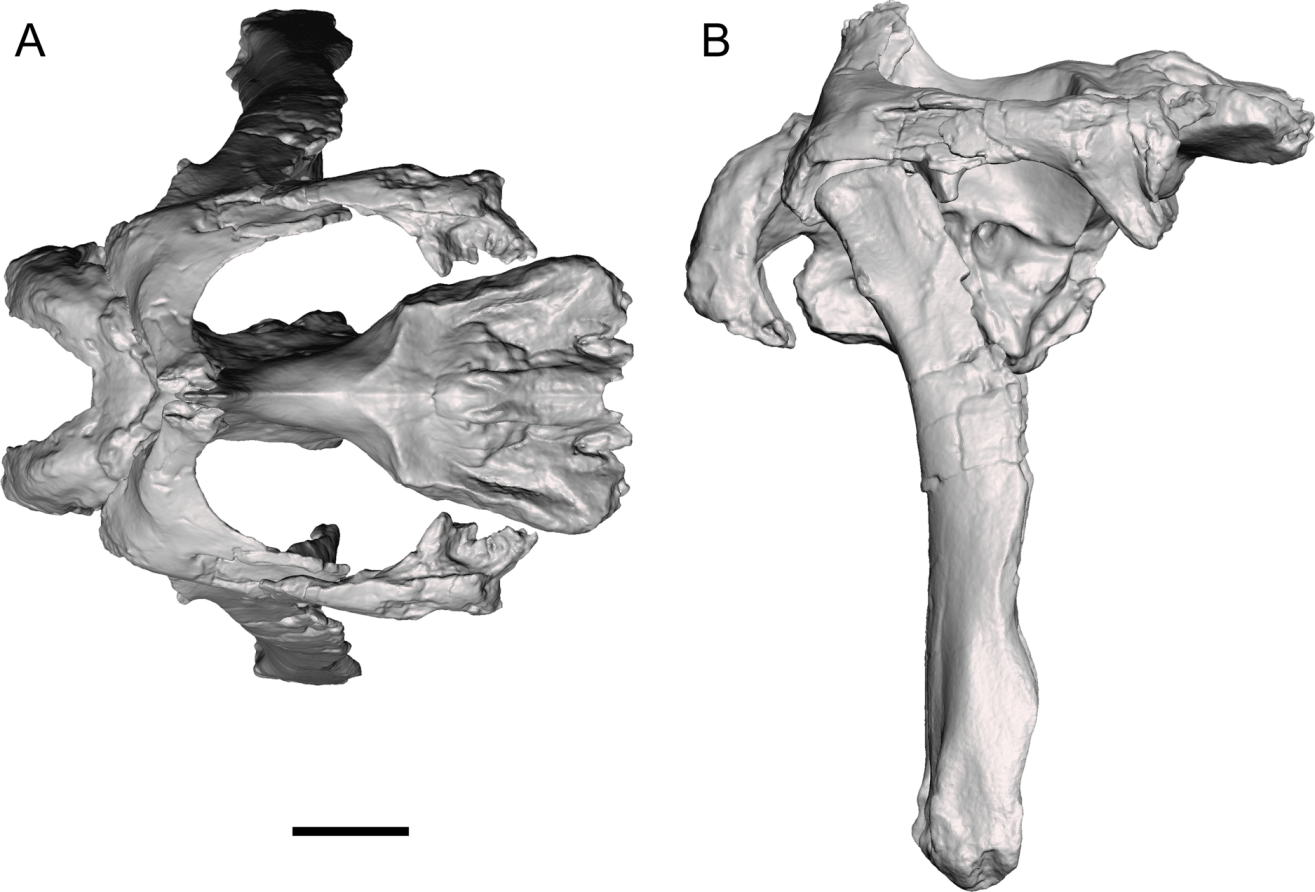
Reconstruction of the caudal region of the skull of WSC 10058, holotype of *Ornatops incantatus*. Orthographic views of a digital 3D model in (A) dorsal and (B) right lateral views. The right half of the braincase, right postorbital, right squamosal, and left quadrate have been mirrored to create this reconstruction. Scale bar equals 5 cm.

The caudally expanded nasofrontal suture of *Ornatops* indicates that it had a transversely broad nasal crest closely appressed to the dorsal surface of the frontals as in *Probrachylophosaurus* and *Brachylophosaurus* (Prieto-Márquez, 2005; Cuthbertson & Holmes, 2010; Freedman Fowler & Horner, 2015), although the exact size and shape of the crest are unknown. A reconstruction of the caudal region of the skull of WSC 10058 closely resembles in general appearance those of *Probrachylophosaurus* (Freedman Fowler & Horner, 2015) and *Brachylophosaurus* (Prieto-Márquez, 2005; Cuthbertson & Holmes, 2010), with large supratemporal fenestrae oriented rostrolaterally to caudomedially, straight temporal bars formed by the postorbitals and squamosals, and tall quadrates (Fig. 12). *Ornatops*, from the Allison Member of the Menefee Formation in New Mexico, is the first crested brachylophosaurin discovered in southern Laramidia. In UMNH VP 16607, a partial skull from the Wahweap Formation in Utah referred to the crestless *Acristavus*, the nasofrontal suture does not extend onto the dorsal surface of the frontals (Gates et al., 2011).

Freedman Fowler & Horner (2015) proposed an anagenetic sequence from the unmodified nasofrontal suture and crestless form of an ancestor like *Acristavus*, to the moderately expanded nasofrontal suture and small crest of *Probrachylophosaurus*, to the greatly expanded nasofrontal suture and large crest of *Brachylophosaurus*. *Ornatops* might be intermediate between the latter two taxa, with a nasofrontal suture expanded farther caudally than in adult *Probrachylophosaurus*, but comparable to subadult *Brachylophosaurus* and less than in adult *Brachylophosaurus*. This scenario implies at least one dispersal event between northern and southern Laramidia. However, the autapomorphic caudally elevated structure and parasagittal bumps on the nasofrontal suture of *Ornatops* are not known to occur in subadult or adult *Brachylophosaurus*; rather, the suture remains horizontal in *Brachylophosaurus*, with the only ontogenetic change to it being increased caudal expansion until it covers the entire dorsal surface of the frontals (Freedman Fowler & Horner, 2015).

Greater sampling of southern brachylophosaurins is necessary to determine the biogeographic and evolutionary history of the lineage or lineages represented by the crested *Ornatops*, *Probrachylophosaurus*, and *Brachylophosaurus*, as well as a framework of absolute dates from the Allison Member to clarify the exact age of *Ornatops*. The crestless *Acristavus* occurs in Montana and Utah (Gates et al., 2011). Perhaps *Probrachylophosaurus* might also be found in southern Laramidia, higher in the Wahweap Formation than *Acristavus*, or lower in the Allison Member than *Ornatops*. Conversely, perhaps *Ornatops* might be discovered in northern Laramidia, higher in the Judith River Formation than *Probrachylophosaurus*. Though current evidence indicates that *Ornatops* is closely related to *Probrachylophosaurus* and *Brachylophosaurus*, it is possible that future discoveries could support alternative scenarios, such as that *Ornatops* evolved the expanded nasofrontal suture convergently and is derived from *Acristavus* in southern Laramidia or an unknown ancestor. Additional fragmentary brachylophosaurin specimens from the Wahweap and Kaiparowits formations of Utah hint at an undiscovered diversity in southern Laramidia (Gates et al., 2013).

## Conclusions

*Ornatops incantatus* is a new genus and species of brachylophosaurin hadrosaurid known from the holotype partial skeleton from the Allison Member of the Menefee Formation in New Mexico. The morphology of the caudally expanded nasofrontal suture in *O*. *incantatus* indicates that it is closely related to and possibly intermediate between *Probrachylophosaurus bergei* and *Brachylophosaurus canadensis*, although additional material is necessary to fully explore the evolution of brachylophosaurins in southern Laramidia. The expanded nasofrontal suture of *O*. *incantatus* indicates the presence of a solid nasal crest, similar to *P*. *bergei* and *B*. *canadensis*, although the shape of the crest is unknown. *O*. *incantatus* is the first crested brachylophosaurin found in southern Laramidia, illustrating the potential of the Menefee Formation to inform future studies on dinosaur evolution in North America during the Campanian.

## Supplemental Information

10.7717/peerj.11084/supp-1 Supplemental Information 1 Data Matrix.

10.7717/peerj.11084/supp-2 Supplemental Information 2 Character List.

10.7717/peerj.11084/supp-3 Supplemental Information 3 Specimen List.

10.7717/peerj.11084/supp-4 Supplemental Information 4 Phylogenetic data matrix. Data matrix was formatted for use with TNT.

## Institutional abbreviations

AEHM: Amur Natural History Museum of the Far Eastern Institute of Mineral Resources
FEB RAS: Blagoveschensk, Russia
AMNH: American Museum of Natural History, New York, New York, USA
ANSP: Academy of Natural Sciences, Philadelphia, Pennsylvania, USA
CEUM: College of Eastern Utah Prehistoric Museum, Price, Utah, USA
CM: Carnegie Museum of Natural History, Pittsburgh, Pennsylvania, USA
CMN: Canadian Museum of Nature, Ottawa, Ontario, Canada
DMNS: Denver Museum of Nature and Science, Denver, Colorado, USA
FMNH: Field Museum of Natural History, Chicago, Illinois, USA
IRSNB: Institut royal des Sciences naturelles de Belgique, Brussels, Belgium
IVPP: Institute of Vertebrate Paleontology and Paleoanthropology, Beijing, China
LACM: Natural History Museum of Los Angeles County, Los Angeles, California, USA
MIWG: Museum of Isle of Wight Geology (Dinosaur Isle Museum), Sandown, UK
MNHN: Muséum national d’Histoire naturelle, Paris, France
MOR: Museum of the Rockies, Bozeman, Montana, USA
MSM: Arizona Museum of Natural History (formerly Mesa Southwest Museum), Mesa, Arizona, USA
MWC: Museum of Western Colorado, Grand Junction, Colorado, USA
NHMUK: The Natural History Museum, London, UK
NMMNH: New Mexico Museum of Natural History and Science, Albuquerque, New Mexico, USA
OTM: Old Trail Museum, Choteau, Montana, USA
PIN: Palaeontological Institute, Moscow, Russia
RAM: Raymond M. Alf Museum of Paleontology, Claremont, California, USA
SDSM: South Dakota School of Mines and Technology, Rapid City, South Dakota, USA
SMU: Southern Methodist University Shuler Museum of Paleontology, Dallas, Texas, USA
TMDC: The Montana Dinosaur Center, Bynum, Montana, USA
TMP: Royal Tyrrell Museum of Paleontology, Drumheller, Alberta, Canada
UMNH: Natural History Museum of Utah (formerly Utah Museum of Natural History), Salt Lake City, Utah, USA
USNM: National Museum of Natural History, Washington, DC, USA
WSC: Western Science Center, Hemet, California, USA
YPM: Yale Peabody Museum of Natural History, New Haven, Connecticut, USA

## Additional Information and Declarations

### Field Study Permissions

The following information was supplied relating to field study approvals (i.e., approving body and any reference numbers): The fossil was collected on land administered by the U.S. Bureau of Land Management, under permit NM 18-03S.

### Data Availability

The following information was supplied regarding data availability: The data, including lists of characters, taxa, and the data matrix used in the phylogenetic analysis, are available in the Supplemental Files. Scans are available at MorphoSource under project ID 1121: - Quadrate: https://doi.org/10.17602/M2/M344144 - Premaxilla: https://doi.org/10.17602/M2/M343803 - Postorbital: https://doi.org/10.17602/M2/M343798 - Squamosal: https://doi.org/10.17602/M2/M343792 - Braincase: https://doi.org/10.17602/M2/M159323 The fossils described in this paper are accessioned at the Western Science Center, Hemet, California, under specimen number WSC 10058.

### New Species Registration

The following information was supplied regarding the registration of a newly described species: Publication LSID: urn:lsid:zoobank.org:pub:BA68A73C-5628-47FB-8EB0-3A8EC54F42EF. *Ornatops incantatus* LSID: urn:lsid:zoobank.org:act:B8A6F41B-0D0B-4D48-8D9B-8CF196458ABB. *Ornatops incantatus* McDonald, Wolfe, Freedman Fowler & Gates LSID: urn:lsid:zoobank.org:act:45E3B696-0FF8-492E-837D-11BB13A81299.
