# Supplementary material for: A new brachylophosaurin (Dinosauria: Hadrosauridae) from the Upper Cretaceous Menefee Formation of New Mexico
Source: PeerJ. 2021 Apr 2;9:e11084. doi: 10.7717/peerj.11084 (PMC8020878; doi:10.7717/peerj.11084)
Supplement: Supplemental Information 2 [file peerj-09-11084-s002.docx]

**S3, Character List and Additional References**

**Character List**

Description of characters used in the phylogenetic analysis of Ankylopollexia. Characters are numbered 0–203 in the format of TNT. Abbreviations refer to specific skeletal elements or regions (e.g., PD1 is predentary character 1): PD, predentary; DT, dentary; SU, surangular; ANG, angular; PM, premaxilla; N, nasal; MX, maxilla; LAC, lacrimal; PRF, prefrontal; PO, postorbital; JG, jugal; QU, quadrate; SQ, squamosal; BC, braincase; T, teeth; AX, axial column; PC, pectoral girdle; FL, forelimb; PV, pelvic girdle; HL, hind limb.

**Mandibular**

0. Predentary, overall shape of oral portion in dorsal view: arcuate, rounded rostrolateral corners (0); subtriangular, comes to a point without a distinct rostral portion (1); subrectangular, squared corners and straight, well demarcated rostral portion (2) (modified from Weishampel et al. 2003, character 18; and Prieto-Márquez et al. 2006b, character 5). PD1

1. Predentary, directions of lateral margins of lateral processes relative to each other in dorsal and ventral views: divergent (0); parallel (1). PD2

2. Predentary, dorsomedial process: present (0); absent (1). PD3

3. Predentary, denticle morphology: large, conical median denticle with one or two prominent conical denticles of subequal size adjacent to the median denticle on both sides and smaller, tab-like denticles on lateral processes (0); rostrocaudally compressed prong-like denticles that increase in size towards the midline of the predentary (1); rostrocaudally compressed prong-like denticles of equal size (2) (modified from Prieto-Márquez 2010b, characters 25 and 27) PD4

4. Dentary, orientation of symphysis relative to lateral margin of dentary: rostrolateral to caudomedial (medial edge of symphysis and lateral margin of dentary diverge caudally in dorsal view) (0); parallel (1) (Prieto-Márquez et al. 2006b, character 10). DT1

5. Dentary, medial or lateral profile of the dorsal margin of the rostral edentulous region of the dentary for articulation with the predentary: pronounced concavity (0); almost straight or straight, or even displaying a subtle convexity (1) (modified from Prieto-Márquez 2010b, character 40, and Godefroit et al. 2012b, character 23). DT2

6. Dentary, diastema: absent (0); present (1). DT3

7. Dentary, shape of tooth row in dorsal view: bowed medially along caudal half (0); straight (1) (modified from Prieto-Márquez et al. 2006b, character 8). DT4

8. Dentary, shape of tooth row in lateral view: straight (0); convex (1). DT5

9. Dentary, orientation of tooth row relative to lateral surface of dentary: convergent rostrally and caudally (0); convergent rostrally and divergent caudally (1). DT6

10. Dentary, morphology of tooth alveoli: alveoli shaped by dentary teeth (0); alveoli with parallel vertical walls (1) (Norman 2002, character 33). DT7

11. Dentary, caudal-most extent of tooth row: medial to coronoid process but still rostral to longitudinal axis of the process (0); caudal to longitudinal axis of the coronoid process but still rostral to the caudal margin of the process (1); caudal to the base of the coronoid process (2) (modified from You et al. 2003a, character 29). DT8

12. Dentary, shape in lateral or medial view: dorsal and ventral margins are parallel (0); deepens rostrally (1) (modified from Norman 2004, character 22). DT9

13. Dentary, morphology of ventral margin of rostral ramus leading to the predentary articulation and symphysis: straight (0); ventral margin inflected ventrally, such that it curves gently towards the predentary articulation and symphysis (1); ventral margin curves dorsally towards symphysis (2). DT10

14. Dentary, bulge along ventral margin directly ventral to the base of the coronoid process: absent (0); present (1) (modified from Prieto-Márquez 2010b, character 41). DT11

15. Dentary, bulge on the lateral surface ventral to the coronoid process that gives rise to the process: absent (0); present (1) (modified from Prieto-Márquez 2010b, character 46). DT12

16. Dentary, platform between the tooth row and the coronoid process: absent, tooth row curves into base of coronoid process (0); present (1) (modified from Norman 2002, character 26). DT13

17. Dentary, orientation of coronoid process: caudally inclined (0); vertical (1); rostrally inclined (2) (modified from Prieto-Márquez et al. 2006b, character 7). DT14

18. Dentary, expansion of dorsal end of coronoid process: absent, unexpanded (0); present, along rostral edge only (1); present, along rostral and caudal edges (2) (modified from McDonald et al. 2010b, character 33). DT15

19. Dentary, expansion of dorsal end of coronoid process, position: absent, unexpanded (0); present, ventral to apex (1); present, at apex (2). DT16

20. Dentary, sharp projection on the caudodorsal surface of the coronoid process: absent (0); present (1) (Xing et al. 2014b, character 51). DT17

21. Surangular, surangular foramen: present (0); absent (1) (modified from Weishampel et al. 1993, character 27). SU1

22. Surangular, external mandibular fenestra: small foramen (“accessory foramen”) on surangular near suture with dentary (0); absent (1) (modified from Kobayashi and Azuma 2003, character 15). SU2

23. Surangular, shape of contact with angular in lateral view: inclined rostrodorsal to caudoventral (0); horizontal (1). SU3

24. Surangular, shape of rostrodorsal process in lateral view: rostrocaudally broad and exposed in lateral view (0); rostrocaudally narrow and strap-like, mostly concealed in lateral view by the caudal margin of the coronoid process (1) (modified from Prieto-Márquez 2010b, character 51). SU4

25. Angular, exposure in lateral view: present, groove on ventral margin of surangular for articulation with angular (0); absent, articulation with surangular occurs on the medial surface of that bone (1) (modified from Norman 2002, character 28). ANG1

**Cranial**

26. Premaxilla, rostral bill margin shape: horseshoe-shaped, forms a continuous semicircle that curves smoothly to postoral constriction (0); subrectangular, square rostral margin (1); broadly arcuate across rostral margin, constricts abruptly behind the oral margin (2) (modified from Evans and Reisz 2007, character 2). PM1

27. Premaxilla, morphology of caudolateral corner of oral margin in lateral view: free and gently curved (0); free and angular (1). PM2

28. Premaxilla, everted rim on lateral edge of oral margin: absent (0); present (1) (modified from Weishampel et al. 1993, character 3). PM3

29. Premaxilla, contour of the rostrolateral region of the thin everted oral margin: everted rim absent (0); broad and arcuate (1); subangular (2) (modified from Prieto-Márquez et al. 2015, character 66). PM4

30. Premaxilla, premaxillary foramen located rostral and ventrolateral to the rostral margin of the apertura ossis nasi: absent (0); present (1) (modified from Horner et al. 2004, character 23, and Prieto-Márquez et al. 2016a, character 50). PM5

31. Premaxilla, denticle morphology: one large conical denticle adjacent to interpremaxillary suture on each premaxilla (0); two large, rostrocaudally elongate denticles on each premaxilla (1); three or more conical denticles of similar size on each premaxilla (2). PM6

32. Premaxilla, morphology of caudolateral process: tapers along entire length (0); dorsoventrally expanded (1) (modified from Prieto-Márquez 2010b, character 71). PM7

33. Premaxilla, transverse ridge of thickened bone caudal to oral margin, separated from the oral margin by a deep sulcus bearing vascular foramina: absent (0); present (1) (modified from Evans and Reisz 2007, character 1, and Prieto-Márquez 2010b, character 63). PM8

34. Premaxilla, shape of external naris: subcircular, with a rounded caudal margin and deeply embayed ventral margin (0); elliptical, with a tapering caudal margin and gently curved ventral margin (1). PM9

35. Premaxilla, external naris composition: bony external naris formed by premaxilla and nasal (0); naris defined entirely by premaxilla, naris elongate (1); lacriform in shape, naris constricted caudally, primarily by lateroventral expansion of caudomedial premaxillary process (2); lacriform in shape, naris constricted caudally primarily by dorsal expansion of caudolateral premaxillary process (3) (modified from Evans and Reisz 2007, character 4). PM10

36. Premaxilla, shape of rostroventral part of narial fossa: floor of fossa oriented dorsomedially and not well exposed in lateral view (0); floor of fossa oriented dorsoventrally and extensively exposed in lateral view (1). PM11

37. Premaxilla, caudal processes (PM1, PM2) and construction of nasal passages: caudomedial premaxillary process short, caudomedial and caudolateral processes do not meet caudal to external nares, nasal passages not enclosed ventrally, external nares exposed in lateral view (0); caudolateral and caudomedial processes elongate and join behind external opening of narial passages to exclude nasals, nasal vestibule completely enclosed by tubular premaxillae, left nasal passage divided from right passage, homologue of iguanodontian external naris not exposed in lateral view (1) (Horner et al. 2004, character 27, modified by Evans and Reisz 2007, character 3, modified again here). PM12

38. Premaxilla, caudolateral process flares dorsally into external naris: absent (0); present (1) (modified from Gates and Sampson 2007, character 31). PM13

39. Premaxilla, orientation of the caudomedial process relative to the caudolateral process around the apertura ossis nasi: subparallel (0); processes slightly converging caudally (1) (modified from Prieto-Márquez et al. 2015, character 67). PM14

40. Premaxilla, vertical groove on the caudolateral process rostral to the maxillary dorsal process that extends ventrally from a small lateral opening between the premaxillary caudal processes: absent (0); present (1) (Evans and Reisz 2007, character 5). PM15

41. Premaxilla, expanded caudally and dorsally to form hollow nasal crest: absent (0); present (1) (modified from Evans and Reisz 2007, character 10). PM16

42. Premaxilla, elongation of the caudolateral process above the prefrontal: absent (0); present (1) (Suzuki et al. 2004, character 4, modified by Evans and Reisz 2007, character 6). PM17

43. Premaxilla, division of circumnarial fossa: circumnarial fossa absent (0); circumnarial fossa tripartite, divided into dorsal fossa, ventral fossa, and accessory rostral fossa by two fine ridges that converge rostroventral to the external bony naris (1); circumnarial depression bipartite, divided into elongate caudal fossa and rostrocaudally narrow rostral fossa (= “outer (accessory) narial fossa”) by strong transverse ridge (2); circumnarial fossa tripartite, divided into elongate caudal fossa, and with rostrocaudally narrow rostral fossa divided into rostrodorsal and rostroventral fossae by triradiate vestibular promontory (3); circumnarial fossa tripartite, divided into three subparallel accessory fossae by two rostrolaterally-extending ridges (4); circumnarial fossa undivided (5) (modified from Gates et al. 2011, character 28, and Prieto-Márquez et al. 2015, character 54; see also Prieto-Márquez and Wagner 2013a and 2013b, and Prieto-Márquez et al. 2016a). PM18

44. Composition of the caudal margin of the functional external naris: formed by the nasal dorsally and the premaxilla ventrally (0); formed entirely by the nasal (1); formed entirely by the premaxilla (2) (Prieto-Márquez et al. 2015, character 165). PM19

45. Circumnarial fossa on the lateral surface of the facial region of the skull: absent, circumnarial structure entirely enclosed (0); present (1) (Horner et al. 2004, character 31). N1

46. Caudodorsal extension of circumnarial fossa: caudal region of fossa absent (0); the fossa extends as far as to surround the caudal margin of the apertura ossis nasi, but is well separated from the orbit (1); the fossa extends caudally such that its caudodorsal region is located dorsal to the rostral half of the lacrimal (2); the fossa extends as far as the rostrodorsal region of the orbit (3); the fossa extends caudodorsal to the orbit (4) (modified from Gates and Sampson 2007, character 36, and Prieto-Márquez et al. 2015, character 167). N2

47. Degree of excavation of the caudal region of the circumnarial fossa: caudal region of fossa absent (0); lightly incised (1); deeply incised (2) (modified from Gates and Sampson 2007, character 37, and Godefroit et al. 2012b, character 113, character treated as ordered). N3

48. Nasal, caudodorsal region of circumnarial fossa well demarcated by thick, arcuate ridge on lateral surface of nasal: absent (0); present (1) (modified in part from Xing et al. 2014b, character 162). N4

49. Nasal, location of the rostral end of the dorsal process of the nasal relative to the rostral margin of the apertura ossis nasi: the rostral end of the dorsal process of the nasal does not reach the rostral margin of the apertura ossis nasi (0); the rostral end of the rostrodorsal process of the nasal reaches the rostral margin of the apertura ossis nasi (1) (Prieto-Márquez et al. 2015, character 72). N5

50. Lateral profile of the apertura ossis nasi: broad and subellipsoidal in lateral profile (0); narrow and subellipsoidal in lateral profile (1); extremely narrow, slit-like in lateral profile (2) (Prieto-Márquez et al. 2015, character 158). N6

51. Nasals, position of nasal cavity: nasals flat caudodorsally and restricted to area rostral to braincase, nasal cavity rostromedial to orbits (0); nasals retracted to lie over braincase in adults (1) (Horner et al. 2004, character 33, modified by Evans and Reisz 2007, character 7, modified again here). N7

52. Nasals, crest arising from caudodorsal surface, invaded by hollow nasal passages: absent (0); present (1) (see Godefroit et al. 2004a). N8

53. Nasals, nasal vestibule morphology, s-loop in the enclosed premaxillary passages rostral to dorsal process of maxilla: absent (0); present (1) (modified from Evans and Reisz 2007, character 8). N9

54. Nasal, caudoventral region of the nasal ventrally recurved and hook-shaped, with a rostral process that inserts under the caudolateral process of the premaxilla: absent (0); present (1) (Prieto-Márquez 2010b, character 80). N10

55. Hollow nasal crest, tubular, elongate, and extends caudally well beyond the occiput, composed largely of the caudomedial processes of the premaxillae: absent (0); present (1) (modified from Sullivan and Williamson 1999, and Evans and Reisz 2007, character 9). N11

56. Hollow nasal crest, length: crest absent or extends beyond occiput less than basal skull length (0); extends posterior to occiput more than basal skull length (1) (Sullivan and Williamson 1999, and Evans and Reisz 2007, character 12). N12

57. Hollow nasal crest, helmet-shaped crest with apex above orbit, nasal forms a large, plate-like portion of the caudal external crest surface: absent (0); present (1) (modified from Evans and Reisz 2007, character 11). N13

58. Hollow nasal crest, procumbent: absent (0); present (1) (modified from Evans and Reisz 2007, character 13). N14

59. Hollow nasal crest, morphology of caudal extremity of caudolateral process of premaxilla: absent (0); expanded, rostral lobe approximately level with or higher than caudal lobe (1); expanded, caudal lobe higher than rostral lobe (2); expanded, rostral and caudal lobes indistinct (3); unexpanded, tapering, and truncated, such that its participation in the crest is limited (4) (modified from Horner et al. 2004, character 35, and Evans and Reisz 2007, character 14 [see also Evans 2010]). N15

60. Hollow nasal crest, enclosure of the nasal passages on lateral crest surface between the caudolateral process of the premaxilla and nasal: premaxilla-nasal fontanelle absent (0); absent, premaxilla-nasal fontanelle present and persists into late ontogeny (1); present, premaxilla-nasal fontanellae completely closed in adult individuals (2) (modified from Evans and Reisz 2007, character 15 [see also Brink et al. 2014]). N16

61. Hollow nasal crest, composition of caudal margin of fan-shaped crest: absent (0); present, composed of premaxilla caudomedial process, which is visible in lateral view (1); present, composed of nasal, nasals have long external internasal joint along caudal and caudoventral margin of crest (2) (modified from Evans and Reisz 2007, character 16). N17

62. Hollow nasal crest, rostral nasal-caudomedial process of premaxilla contact: absent (0); rostrodorsal margins of nasals form rhomboid processes that overlap premaxilla caudomedial processes laterally (1); present, rostral end of nasal fits along ventral edge of premaxilla (2); present, premaxilla and nasal meet in a complex W-shaped interfingering suture in which a long, finger-like process of the nasal has an extensive overlapping joint with the caudomedial process of the premaxilla in the rostral region of the crest (3); nasal fits into a dorsoventrally elongate facet on the caudal margin of the caudomedial process of the premaxilla (4) (modified from Evans and Reisz 2007, character 17). N18

63. Hollow nasal crest, premaxilla caudomedial process with accessory rostroventral flange that overlaps the lateral surface of the nasal in the rostral region of the crest: absent (0); present (1) (Evans and Reisz 2007, character 18). N19

64. Solid nasal crest, association with caudal margin of circumnarial fossa: solid nasal crest absent (0); solid crest present but circumnarial fossa does not excavate side of crest, fossa terminates rostral to solid crest (1); solid crest present, excavated laterally by circumnarial fossa (2) (modified from Gates et al. 2011, character 37). N20

65. Solid nasal crest, caudal ends of paired nasals form transversely-wide crest over snout or braincase: absent (0); present (1) (modified from Gates et al. 2011, character 36). N21

66. Solid nasal crest, transversely-wide crest paddle-shaped, extending caudally to overhang the parietal in adults: absent (0); present (1) (see Freedman Fowler and Horner 2015). N22

67. Solid nasal crest, creating a mediolaterally compressed arch rostral to orbits: absent (0); present (1) (Gates and Sampson 2007, character 42). N23

68. Solid nasal crest, position of the summit of solid mediolaterally compressed arch crest relative to the caudodorsal margin of the apertura ossis nasi: absent (0); summit located dorsal to the caudal margin of the apertura ossis nasi (1); summit located caudodorsal to the caudal margin of the apertura ossis nasi (2) (modified from Prieto-Márquez et al. 2015, character 77). N24

69. Solid nasal crest deeply excavated laterally by the circumnarial fossa: absent (0); present (1) (see McGarrity et al. 2013 and Prieto-Márquez et al. 2015). N25

70. Solid nasal crest deeply excavated laterally by the circumnarial fossa, morphology: absent (0); caudally-recurved eminence rostrodorsal to orbits (1); dorsally-curved eminence rostrodorsal to orbits (2); significantly elevated dorsally above the skull roof, with bluntly subconical distal end (3) (modified from Prieto-Márquez et al. 2015, characters 176 and 177). N26

71. Maxilla, rostral projection of rostrodorsal process: not visible in lateral view of external nares (0); projects into external nares in lateral view due to elongation of rostrodorsal process (1); projects into external nares in lateral view due to down-turning of premaxilla (2) (modified from Gates et al. 2011, character 40). MX1

72. Maxilla, rostrodorsal region expanded to form prominent subrectangular flange that rises vertically above rostroventral process: absent (0); present (1) (see Prieto-Márquez et al. 2013). MX2

73. Maxilla, direction of rostroventral process: rostrally directed (0); rostroventrally curved (1). MX3

74. Maxilla, ventral margin of tooth row in lateral view: straight (0); concave (1). MX4

75. Maxilla, shape in dorsal view: bowed medially (0); straight for most of length (1); bowed laterally (2). MX5

76. Maxilla, shape of tooth row in ventral view: medially bowed, with rostral and caudal ends curving laterally (0); bowed laterally (1); straight (2). MX6

77. Maxilla, jugal process morphology: sinuous shelf, scarf contact with jugal (0); caudolaterally projecting jugal process, “finger-in-recess” contact with jugal (1); mediolaterally broad promontory located dorsal and rostral to the ectopterygoid shelf, bearing a concave and subtriangular dorsolaterally-facing surface against which jugal abuts (2); subtriangular joint surface for the jugal that is more laterally than dorsally facing, with a lateroventrally-directed pointed corner that is located adjacent and slightly dorsal to the proximal end of the lateral ridge of the ectopterygoid shelf (3); dorsally elevated jugal joint (distance between the ventral margin of the jugal joint and ectopterygoid shelf nearly equal to depth of the caudal segment of the maxilla), caudal margin of the joint flush with the caudal margin of the rostrodorsal eminence of the lateral side of the maxilla (4) (modified from Norman 2002, character 15, and Prieto-Márquez 2010b, character 92). MX7

78. Maxilla, antorbital fossa, extent in lateral view: occupies most of lateral surface of ascending process (0); rostrocaudally elongate, elliptical depression restricted to caudal half of ascending process (1); small semicircular depression restricted to caudal margin of ascending process (2); antorbital fossa not visible in lateral view (3). MX8

79. Maxilla, bifurcation of maxillary ascending process into lateral and medial lacrimal processes: absent (0); present (1). MX9

80. Maxilla, morphology of rostrodorsal process: absent (0); present, low eminence on rostrodorsal margin of maxilla (1); present, prominent rostrally-projecting prong separated from the rostroventral process by a deep embayment (2); rostrodorsal margin of maxilla forms a sloping shelf that underlies the premaxilla (3) (modified from Prieto-Márquez et al. 2006b, character 17, and Evans and Reisz 2007, character 19). MX10

81. Maxilla, ascending process shape in lateral view: low and gently rounded (0); tall and sharply peaked (1) (modified from Evans and Reisz 2007, character 20). MX11

82. Maxilla-lacrimal contact: present externally (0); largely covered externally by a jugal-premaxilla contact (1) (Evans and Reisz 2007, character 23). MX12

83. Maxilla, ectopterygoid ridge: poorly developed (0); strongly developed, thickened horizontal ridge on lateral surface of maxilla (1) (Evans and Reisz 2007, character 24). MX13

84. Lacrimal, concave ventral margin to form part of antorbital fenestra: present (0); absent (1). LAC1

85. Lacrimal, morphology of rostral ramus: tapers to a point (0); dorsoventrally expanded (1). LAC2

86. Lacrimal, morphology of ventral ramus: tapers to a point (0); rounded (1). LAC3

87. Lacrimal, contact with nasal: present (0); absent (1) (Norman 2002, character 12). LAC4

88. Prefrontal, morphology of nasal process: tapering, finger-like projection (0); dorsoventrally broad, mediolaterally compressed plate (1). PRF1

89. Prefrontal, medial margin forms a thin vertical flange that laps onto the base of the crest: absent (0); present (1) (Evans and Reisz 2007, character 32). PRF2

90. Prefrontal, medial flange extends caudally over the dorsal surface of the frontal and above the prefrontal-postorbital joint in lateral view (in adults): absent (0); present (1) (Evans and Reisz 2007, character 33). PRF3

91. Prefrontal, caudodorsal process that joins the caudodorsal frontal process and participates in the lateroventral portion of a solid crest: absent (0); present (1) (modified from Bell 2011a, character 22, and Prieto-Márquez et al. 2015, character 118). PRF4

92. Postorbital, shape of caudal end of squamosal process that overlaps the lateral surface of the squamosal: rounded (0); bifurcated (1) (modified from Prieto-Márquez 2010b, character 132). PO1

93. Postorbital, gentle protuberance on dorsal surface near the rostral end of the squamosal process: absent (0); present (1) (see Prieto-Márquez et al. 2016b). PO2

94. Postorbital, length of squamosal process: postorbital-squamosal contact does not reach caudal margin of supratemporal fenestra (0); contact reaches the caudal margin of the supratemporal fenestra (1) (modified from Prieto-Márquez et al. 2006b, character 37). PO3

95. Postorbital, dorsal ‘promontorium’: absent (0); present in adults (1) (Evans and Reisz 2007, character 37). PO4

96. Postorbital, strongly inflected (Y-shaped) dorsal surface of the central body of the postorbital (in presumably adult larger specimens): absent (0); present (1) (Prieto-Márquez et al. 2015, character 124). PO5

97. Postorbital, morphology of jugal process of the postorbital: relatively robust in lateral view, strongly expanding rostroventrally along the rostral margin of its dorsal half (0); slender, gradually tapering ventrally (1); rostrocaudally broad in lateral profile, strongly expanded along its rostral and caudal margins (2) (modified from Xing et al. 2014b, character 182). PO6

98. Postorbital, deep fossa (“postorbital pocket”) along orbital margin: absent (0); present (1) (modified in part from Xing et al. 2014b, character 183). PO7

99. Jugal, articulation with ectopterygoid: present (0); absent (1) (Head 1998, character 6). JG1

100. Jugal, morphology of the medial articular surface of the rostral process of the jugal: deep concavity roofed by a narrow horizontal shelf (0); deep concavity roofed by an oblique

(rostrodorsally oriented) narrow shelf (1); medial articular surface bounded caudally by a vertical rim of bone (2) (Prieto-Márquez et al. 2016a, character 100). JG2

101. Jugal, morphology of portion of maxillary process that overlaps maxilla: tapers at rostral ends of maxillary and lacrimal contacts, with slightly convex ventral margin and slightly concave dorsal margin (0); tapers with sinuous dorsal and ventral margins (1); dorsoventrally expanded (2); dorsoventrally expanded to form part of rostral margin of orbit, lacrimal pushed dorsally to lie completely above the level of the maxilla (3) (modified from Norman 2002, character 14, and Evans and Reisz 2007, character 27). JG3

102. Jugal, rostrally-pointed maxillary process: absent (0); present, process restricted to dorsal portion of jugal, rostral jugal appears asymmetrical (1); present, process centered on rostral jugal, rostral jugal appears symmetrically triangular in shape (2) (modified from Gates et al. 2011, character 52). JG4

103. Jugal, morphology of the triangular caudoventral expansion of the rostral process of the jugal: no expansion (0); shallow and rostrocaudally wide prominence (wider than deep) (1); ventrally pointed, approximately as deep as or slightly deeper as its proximal end is wide (2); ventrally projected triangular narrow process, at least twice as deep as it is wide, sharply pointed and often recurved caudally (3) (Godefroit et al. 2012b, character 68). JG5

104. Jugal, large neurovascular foramen at base of postorbital process on medial surface: absent (0); present (1). JG6

105. Jugal, shape of free ventral margin caudal to maxillary contact: sinuous, jugal dorsoventrally expanded ventral to infratemporal fenestra (0); sinuous with striated, caudally-directed flange that projects caudal to jugal-quadratojugal contact (1); angular, with prominent ventrally-directed flange ventral to infratemporal fenestra (2); dorsoventrally narrow and strap-like, with convex ventral margin and concave dorsal margin that are parallel (3); angular, jugal dorsoventrally constricted beneath infratemporal fenestra to set off flange rostral to constriction (4) (modified from Norman 2002, character 16, and Evans and Reisz 2007, character 30). JG7

106. Jugal, ventral flange size, ratio of depth of jugal at constriction below infratemporal fenestra to length of free ventral flange on jugal: ventral flange absent (0); small (1); prominent, well set off from jugal body, smooth and gently arching along entire caudoventral margin (2); prominent, well set off from jugal body, caudoventral margin slightly angular as if “pulled” down (3) (modified from Gates et al. 2011, character 55). JG8

107. Jugal, articulation with jugal process of postorbital: postorbital process of jugal notched at dorsal end (0); postorbital process of jugal bears an elongated facet on its cranial margin (1) (modified from Head 1998, character 7). JG9

108. Jugal, relative widths of the orbital and infratemporal margins in lateral view: orbital margin wider (0); infratemporal margin wider (1) (modified from Prieto-Márquez 2010b, character 115, and Sullivan et al. 2011, character 32). JG10

109. Quadrate, shape of notch in lateral wing: semicircular (0); broad and crescentic (1) (modified from Prieto-Márquez et al. 2006b, character 40). QU1

110. Quadrate, overall shape in lateral or medial view: straight for much of dorsoventral length, curved caudally near dorsal end (0); curved gently caudally along entire length (1); straight (2). QU2

111. Quadrate, shape of dorsal condyle: subtriangular, broad rostral margin and tapers to a point caudally (0); D-shaped, broadest along lateral profile (1) QU3

112. Quadrate, shape of ventral condyle: rostrocaudally narrow and mediolaterally broad, with larger lateral condyle, and medial condyle slopes ventrolaterally towards lateral condyle (0); distinct step between medial condyle and larger, more ventrally situated lateral condyle (1). QU4

113. Quadrate, shape of quadrate buttress: low eminence that grades into dorsal condyle (0); prominent flange well set off from shaft of quadrate and dorsal condyle (1) (see Freedman Fowler and Horner 2015). QU5

114. Squamosal, orientation of caudomedial process: curved rostromedially (0); straight and medially directed (1) (modified from Prieto-Márquez et al. 2006b, character 45). SQ1

115. Squamosal, relationship of right and left squamosals on skull roof: widely separated by parietal (0); separated by only a narrow band of the parietal (1); in broad contact with each other, parietal excluded from occipital aspect of skull (2) (modified from Godefroit et al. 2001, character 3, and Horner et al. 2004, character 63). SQ2

116. Squamosal, shape of caudoventral surface: shallowly exposed in caudal view (0); forms a deep, near vertical, well-exposed face in caudal view (1) (Evans and Reisz 2007, character 48). SQ3

117. Squamosal, height above quadrate cotylus: lateral side relatively low (0); markedly expanded dorsally above the cotylus (1) (Evans and Reisz 2007, character 49). SQ4

118. Frontal, participation in dorsal orbital rim: present (0); absent (1) (Norman 2002, character 19). BC1

119. Frontal, upward doming over braincase in juveniles and/or adults: absent (0); present (1) (modified from Horner et al. 2004, character 58). BC2

120. Frontal, platform for nasal articulation: absent (0); present, comprised of two thin, rostroventrally-curved tongues that are widely separated along the midline and form the nasal articulation surface with ectocranial processes of frontals (1); present, comprised of two thin, rostroventrally-curved tongues that meet along the midline of the skull roof and form a median cleft (2); present, thickened and steeply angled, with median cleft absent (3) (modified from Evans and Reisz 2007, character 40; see also Prieto-Márquez and Wagner 2013b). BC3

121. Frontal, morphology of nasofrontal suture: no distinct processes (0); nasofrontal suture is transversely wide corrugated structure that extends caudally to cover more than half of the dorsal surface of the frontals in adults (1); paired nasals insert small process between rostromedial margins of paired frontals (2); process along rostral margin of paired frontals inserts between bifurcated nasals, forming a W-shaped suture (3); frontals rise caudodorsally to support solid nasal crest (4) (modified from Gates and Sampson 2007, character 65, based upon McGarrity et al. 2013). BC4

122. Frontal, nasal articulation surface extends caudodorsally to overhang the supratemporal fenestrae in adults: absent (0); present (1) (modified from Godefroit et al. 2001, character 5, and Evans and Reisz 2007, character 41). BC5

123. Frontal, caudodorsal process rising from the ectocranial surface of the frontal to buttress the ventral surface of nasal crest: absent (0); present (1) (Prieto-Márquez et al. 2015, character 139). BC6

124. Frontal, shape of ectocranial surface: elongate with an ectocranial length/width > 0.8 (0); relatively short, with a length/width < 0.8 (1); greatly shortened length/width ratio < 0.4) (2) (Evans and Reisz 2007, character 42). BC7

125. Laterosphenoid, complete enclosure of ophthalmic sulcus by bone laterally: absent (0); present (1) (Evans and Reisz 2007, character 51). BC8

126. Laterosphenoid, great reduction of the length of the postorbital process: absent (0); present (1) (Xing et al. 2014b, character 221). BC9

127. Supraoccipital, contribution to foramen magnum: present (0); absent, excluded by exoccipitals (1) (You et al. 2003a, character 23). BC10

128. Supraoccipital, morphology of supraoccipital-exoccipital contact: straight suture that meets squamosal (0); ventrolateral corner of supraoccipital is inset into exoccipital so that supraoccipital is locked between exoccipitals (1) (Horner et al. 2004, character 66). BC11

129. Supraoccipital, inclination of caudal surface: caudal surface rostrally inclined (0); caudal surface vertical (1) (modified from Horner et al. 2004, character 65). BC12

130. Exoccipital-Opisthotic, paroccipital process orientation of pendant distal portion: straight and ventrally directed (0); curved rostrally (1) (Horner et al. 2004, character 62). BC13

131. Basioccipital, rostrocaudally directed groove extending along ventral surface: present (0); absent (1). BC14

132. Basioccipital, morphology of surface between basal tubera: broad, shallow trough with ridge extending rostrocaudally down the midline (0); broad, shallow trough with smooth floor (1). BC15

133. Basisphenoid, surface between basipterygoid processes: transverse, sharply defined ridge between basipterygoid processes (0); ventrally directed prong between basipterygoid processes (1) (modified from Gates and Sampson 2007, characters 78 and 79). BC16

134. Basisphenoid, alar process morphology: moderate in size (0); large and prominent (1) (Gates et al. 2011, character 75). BC17

135. Foramen magnum, composition of ventral margin: caudomedial surfaces of left and right exoccipitals and dorsal margin of basioccipital (0); left and right exoccipitals only (1) (modified from Weishampel et al. 1993, character 24). BC18

136. Parietal, shape of sagittal crest in lateral view: approximately level with the skull roof (0); deepens caudally (1) (modified from Prieto-Márquez 2010b, character 148). BC19

137. Parietal, rostral extent of the sagittal crest: extends along entire length of parietal and is sharply-defined at rostral end (0); extends along entire length of parietal but diminishes near rostral end (1); extends along only the caudal half of the parietal (2) (modified from Prieto-Márquez 2010b, character 150). BC20

138. Parietal, morphology of median rostral process of the parietal along the frontoparietal suture: arcuate or subtriangular, rostrocaudally short and mediolaterally wide (0); finger-shaped or subtriangular, rostrocaudally long and mediolaterally narrow (1) (modified from Xing et al. 2014b, character 204). BC21

139. Basioccipital, distinct neck between basal tubera and occipital condyle: present (0); absent (1) (Godefroit et al. 2009). BC22

140. Infratemporal fenestra, shape: subrectangular, dorsal margin approximately as wide as the ventral margin (0); subtriangular, dorsal margin narrower than the ventral margin (1) (modified from Prieto-Márquez et al. 2015, character 182). BC23

141. Supratemporal fenestra, shape in dorsal view: oval, long axis directed rostrally (0); oval, long axis directed rostrolaterally (1); oval, long axis oriented mediolaterally (2) (modified from Prieto-Márquez 2010b, character 193). BC24

**Dentition**

142. Dentary teeth, morphology of marginal denticles: tongue-shaped with smooth edges (0); tongue-shaped with mammillated edges (1); reduced to small mammillated papillae, but still distinct (2) absent or extremely faint (3) (modified from Norman 2002, character 31). T1

143. Dentary teeth, number of replacement teeth per tooth position: one (0); two (1); three (2) (modified from Weishampel et al. 1993, character 32). T2

144. Dentary teeth, number of teeth per tooth position forming part of occlusal plane: one (0); two (1); three (2) (modified from Norman 2002, character 39). T3

145. Dentary teeth, shape of crown in lingual view: mesiodistally broad, oblong, shield-like surface (0); mesiodistally narrow and diamond-shaped (1) (modified from Norman 2002, character 29). T4

146. Dentary teeth, position of primary ridge: distally offset (0); no offset, primary ridge divides the lingual side of the crown into equal halves (1) (modified from You et al. 2003a, character 39). T5

147. Dentary teeth, number and morphology of ridges on lingual surface of crown: parallel and similarly prominent primary and secondary ridges with multiple faint accessory ridges arising from marginal denticles (0); prominent primary ridge and multiple separate faint accessory ridges to either side of it (1); primary ridge and a single less prominent accessory ridge on either side (2); primary ridge and a single mesial accessory ridge (3); primary ridge only (4). T6

148. Maxillary teeth, number of teeth per tooth position forming part of occlusal plane: one (0); two (1). T7

149. Maxillary teeth, primary ridge position and morphology: distally offset (0); no offset, primary ridge divides the labial side of the crown into equal halves (1) (modified from You et al. 2003a, character 36). T8

150. Maxillary teeth, number and morphology of ridges on labial surface of crown: primary ridge with multiple parallel accessory ridges on either side (0); primary ridge and only mesial accessory ridges (1); primary ridge only (2). T9

**Postcranial (Axial)**

151. Axis, morphology of axial neural spine in lateral view: caudodorsally-sloping process (0); dorsally expanded, convex process (1). AX1

152. Cervical vertebrae, opsisthocoely of centra: slightly opisthocoelous, flat or slightly convex cranial face (0); deeply opisthocoelous, hemispherical cranial face protrudes beyond ventral and dorsal surfaces of centrum and has a smooth, rounded surface (1). AX2

153. Cervical vertebrae, number: 13 or fewer (0); more than 13 (1) (Evans and Reisz 2007, character 66). AX3

154. Dorsal vertebrae, middle and caudal dorsals, extremely tall neural spines, approximately four times centrum height: absent (0); present (1) (modified from Norman 2002, character 41, and Evans and Reisz 2007, characters 67 and 68). AX4

**Postcranial (Appendicular)**

155. Sternal, caudolateral process: absent (0); present (1) (modified from Kobayashi and Azuma 2003, character 23). PC1

156. Sternal, caudomedial process: absent (0); present (1). PC2

157. Scapula, dorsal margin of scapular shaft at approximately mid-shaft between acromion process and caudodorsal margin: straight (0); convex (1). PC3

158. Scapula, expansion of caudal end: gently convex expansion along caudodorsal margin, caudoventral margin tapers into hook-like flange (0); caudal end paddle-shaped, dorsal and ventral margins of scapula diverge towards caudal end (1); caudal margin of scapula straight, dorsal and ventral margins are parallel approaching caudal margin of scapula and meet caudal margin at nearly right angles (2). PC4

159. Scapula, acromion process orientation: dorsally directed (0); laterally directed (1) (Norman 2002, character 44). PC5

160. Scapula, morphology of deltoid ridge: dorsoventrally narrow with a poorly demarcated ventral margin, restricted to proximal part of scapula (0); dorsoventrally narrow and sharply defined, with a well-demarcated ventral margin (1); dorsoventrally deep and craniocaudally elongated, with a well-demarcated ventral margin (2) (modified from Prieto-Márquez 2010b, character 218). PC6

161. Coracoid, shape of cranial margin: convex (0); straight (1) (modified from Horner et al. 2004, character 78). PC7

162. Coracoid, biceps tubercle size: tubercle small (0); large, laterally projecting biceps tubercle (1) (Evans and Reisz 2007, character 73). PC8

163. Coracoid, cranioventral process (=cranioventral hook): short and weakly developed (0); long, extends well below the glenoid (1) (Evans and Reisz 2007, character 74). PC9

164. Humerus, shape of deltopectoral crest: distal margin rounded and merges gradually with the lateral margin of the humeral shaft (0); distal margin angular and merges abruptly with the lateral margin of the humeral shaft (1) (modified from Weishampel et al. 1993, character 37). FL1

165. Humerus, deltopectoral crest length: short, much less than half the length of the humerus (0); extends at least to midshaft or farther (1) (modified from Evans and Reisz 2007, character 76). FL2

166. Humerus, distal condyles: compressed mediolaterally, flare little from shaft of humerus (0); mediolaterally broad, flare moderately from shaft of humerus (1) (Evans and Reisz 2007, character 78). FL3

167. Manus, digit I: present (0); absent (1) (Norman 2002, character 51). FL4

168. Manus, arrangement of metacarpals II-IV: spreading (0); closely appressed (1) (You et al. 2003a, character 49). FL5

169. Manus, unguals of digits II and III, shape: claw-like (0); flattened and hoof-like (1) (Norman 2002, character 53). FL6

170. Ilium, preacetabular process, cranial end: rounded (0); dorsoventrally-expanded boot offset from shaft (1). PV1

171. Ilium, dorsal margin above pubic and ischial peduncles and acetabulum: straight (0); convex (1); sinuous, convex above pubic peduncle and concave above ischial peduncle (2) (modified from Weishampel et al. 2003, character 55). PV2

172. Ilium, morphology of dorsal margin of postacetabular process dorsal to ischial peduncle: mediolaterally thickened dorsal margin compared to dorsal margin above pubic peduncle (0); thickened and laterally-bulging everted rim along dorsal margin (1); dorsal margin thickened and expanded ventrolaterally to form rounded knob (2); dorsally-projecting flange extending from above acetabulum to above ischial peduncle (3); laterally-projecting, non-pendant supraacetabular process continuous with dorsal margin of ilium (4); pendant supraacetabular process continuous with dorsal margin of ilium (5) (modified from Norman 2002, character 56). PV3

173. Ilium, postacetabular process, shape in lateral view: tapers to a point with break in slope along dorsal margin, forming a distinct platform for the origin of *M. iliocaudalis* (0); tapers with no break in slope along dorsal margin (1); tapers, process curves dorsally along its entire length, such that both the dorsal and ventral margins curve dorsally (2); subrectangular with no break in slope (3) (modified from Norman 2002, character 57). PV4

174. Ilium, curvature of preacetabular process near its base: curves ventrally, with a convex dorsal margin and concave ventral margin (0); straight, with little or no change in slope between its dorsal margin and the dorsal margin of the body of the ilium (1). PV5

175. Ilium, preacetabular process projects ventral to the pubic peduncle: absent (0); present (1). PV6

176. Ilium, morphology of the pubic peduncle: cranioventrally-directed, craniocaudally narrow process (0); craniocaudally broad, subtriangular prominence (1) (modified from Prieto-Márquez 2010b, character 241). PV7

177. Ilium, shape of acetabulum: deep, semicircular, dorsal margin strongly arched (0); shallow and crescentic (1). PV8

178. Ilium, morphology of ischial peduncle: ventrolaterally-directed oval prominence (0); oval prominence with smaller prominence on the caudodorsal margin (1); composed of two prominences of similar size (2) (modified from Prieto-Márquez 2010b, character 242). PV9

179. Ilium, position of ventral-most point on supraacetabular process relative to the caudal-most point on the ischial peduncle: caudal (0); dorsal (1); cranial (2) (modified from Prieto-Márquez 2010b, character 235). PV10

180. Pubis, distal expansion of cranial pubic process: absent, dorsal and ventral margins parallel (0); present, dorsal and ventral margins diverge distally (1) (modified from Norman 2002, character 58). PV11

181. Pubis, caudal pubic process: approximately equal in length to ischium (0); shorter than ischium (1) (Norman 2002, character 59). PV12

182. Pubis, caudal pubic process, morphology of distal end: rounded (0); tapers to a point (1). PV13

183. Pubis, shape of dorsoventral expansion of cranial pubic process: unexpanded (0); asymmetrical, dorsal portion more expanded than ventral, expansion is directed craniodorsally (1); asymmetrical, ventral portion more expanded than dorsal, expansion is directed cranioventrally (2); symmetrical, dorsal and ventral portions approximately equal in size (3) (modified from Prieto-Márquez 2010b, character 252). PV14

184. Pubis, craniocaudal length of the proximal constriction of the cranial pubic process relative to the length of the dorsoventral expansion: unexpanded (0); constriction is longer (1); approximately the same length (2); constriction is shorter (3) (modified from Prieto-Márquez 2010b, character 255). PV15

185. Pubis, shape of proximal constriction of expanded cranial pubic process: unexpanded (0); dorsal and ventral margins parallel (1); maximum ventral concavity located more proximal than maximum dorsal concavity (2); maximum ventral concavity located approximately ventral to the maximum dorsal concavity (3) (modified from Prieto-Márquez 2010b, character 256). PV16

186. Pubis, obturator foramen: present, enclosed by ischial peduncle and caudodorsal process on caudal pubic process (0); absent, no caudodorsal process on caudal pubic process (1) (modified from Prieto-Márquez 2010b, character 258). PV17

187. Ischium, morphology of shaft: curved cranially (0); curved caudally (1); straight (2) (modified from Norman 2002, character 60; Weishampel et al. 2003, character 60). PV18

188. Ischium, morphology of distal end: rounded expansion (0); cranially expanded boot (1); bluntly truncated (2) (modified from Prieto-Márquez et al. 2006b, character 126). PV19

189. Ischium, alignment of long axis of shaft: aligned with bisector between the pubic and iliac peduncles (0); aligned with pubic peduncle (1) (Gasca et al. 2014). PV20

190. Ischium, relative proximodistal lengths of the pubic and iliac peduncles in lateral view: pubic peduncle longer (0); iliac peduncle longer (1); lengths subequal (2) (Gasca et al. 2014). PV21

191. Ischium, relative craniocaudal depths of pubic and iliac peduncles in lateral view: iliac peduncle deeper (0); pubic peduncle deeper (1); depths subequal (2) (Gasca et al. 2014). PV22

192. Ischium, acetabular margin in lateral view: broad and crescentic (0); narrow, U-shaped (1) (modified from Gasca et al. 2014). PV23

193. Ischium, curvature between iliac peduncle and shaft of ischium: gentle curve (0); nearly right-angle (1) (modified from Gasca et al. 2014). PV24

194. Ischium, lip projecting from caudal margin of the articular surface of the iliac peduncle: absent (0); present (1). PV25

195. Femur, curvature of shaft in lateral or medial view: distal half of shaft curved caudally (0); distal half of shaft straight (1) (Norman 2002, character 62). HL1

196. Femur, groove on caudal aspect of femoral head: present (0); absent (1) (Winkler et al. 1997, character 25). HL2

197. Femur, morphology of fourth trochanter: pendant (0); broad and triangular (1); curved, mediolaterally compressed eminence (2) (Norman 2002, character 63). HL3

198. Femur, intercondylar extensor groove: deep, narrow, U-shaped, partially enclosed by slight expansion of medial condyle (0); deep, U-shaped, partially enclosed by expansion of medial and lateral condyles (1); canal fully enclosed by lateral and medial condyles (2) (modified from Norman 2002, character 64; Barrett et al. 2011, character 127). HL4

199. Femur, deep cleft separating the greater and cranial trochanters: present (0); absent, lesser trochanter is closely appressed to the proximal end of the femur (1) (modified from Barrett et al. 2011, character 134). HL5

200. Tibia, shape and extent of cnemial crest on cranial margin: cranially-expanded subtriangular flange restricted to proximal end of tibia (0); further extended along the cranial surface of the proximal half of the diaphysis (1) (modified from Prieto-Márquez 2010b, character 277). HL6

201. Tarsus, modified with cranial ascending process of astragalus equilateral in shape and expanded distal end of fibula: absent (0); present (1) (modified from Evans and Reisz 2007, character 92). HL7

202. Pes, prominent extensor processes extending proximodorsally from the dorsal margins of the proximal articulation facets of the distal phalanges: present (0); absent (1). HL8

203. Pes, morphology of unguals on digits II-IV: dorsoventrally flattened, but elongate and pointed (0); dorsoventrally flattened and elongate, but with blunt truncated tips (1); hoof-like shape (2) (modified from Norman 2002, character 67). HL9

**References**

Barrett, P. M., Butler, R. J., Wang X.-L., and Xu X. 2009. Cranial anatomy of the iguanodontoid ornithopod *Jinzhousaurus yangi* from the Lower Cretaceous Yixian Formation of China. *Acta Palaeontologica Polonica* 54: 35-48.

Barrett, P. M., Butler, R. J., Twitchett, R. J., and Hutt, S. 2011. New material of *Valdosaurus*

*canaliculatus* (Ornithischia: Ornithopoda) from the Lower Cretaceous of southern England. *Special Papers in Palaeontology* 86: 131-163.

Bell, P. R. and Evans, D. C. 2010. Revision of the status of *Saurolophus* (Hadrosauridae) from California, USA. *Canadian Journal of Earth Sciences* 47: 1417-1426.

Bell, P. R. 2011a. Redescription of the skull of *Saurolophus osborni* Brown 1912 (Ornithischia: Hadrosauridae). *Cretaceous Research* 32: 30-44.

Bell, P. R. 2011b. Cranial osteology and ontogeny of *Saurolophus angustirostris* from the Late Cretaceous of Mongolia with comments on *Saurolophus osborni* from Canada. *Acta Palaeontologica Polonica* 56: 703-722.

Bertozzo, F., Dalla Vecchia, F. M., and Fabbri, M. 2017a. The Venice specimen of *Ouranosaurus nigeriensis* (Dinosauria, Ornithopoda). *PeerJ* 5: e3403.

Bertozzo, F., Dal Sasso, C., Fabbri, M., Manucci, F., and Maganuco, S. 2017b. Redescription of a remarkably large *Gryposaurus notabilis* (Dinosauria: Hadrosauridae) from Alberta, Canada. *Memorie della Società Italiana di Scienze Naturali e del Museo Civico di Storia Naturale di Milano* 43: 3-56.

Bolotsky, Y. L. and Godefroit, P. 2004. A new hadrosaurine dinosaur from the Late Cretaceous of far eastern Russia. *Journal of Vertebrate Paleontology* 24: 351-365.

Bolotsky, Y. L., Godefroit, P., Bolotsky, I. Y., and Atuchin, A. 2014. Hadrosaurs from the Far East: historical perspective and new *Amurosaurus* material from Blagoveschensk (Amur Region, Russia). In *Hadrosaurs* (eds. D. Evans and D. Eberth), pp. 315-331. Bloomington: Indiana University Press.

Boyd, C. A. and Pagnac, D. C. 2015. Insight on the anatomy, systematic relationships, and age of the Early Cretaceous ankylopollexian dinosaur *Dakotadon lakotaensis*. *PeerJ* 3: e1263.

Brill, K. and Carpenter, K. 2006. A description of a new ornithopod from the Lytle Member of the Purgatoire Formation (Lower Cretaceous) and a reassessment of the skull of *Camptosaurus*. In *Horns and Beaks: Ceratopsian and Ornithopod Dinosaurs* (ed. K. Carpenter), pp. 49-67. Bloomington: Indiana University Press.

Brink, K. S., Zelenitsky, D. K., Evans, D. C., Therrien, F., and Horner, J. R. 2011. A sub-adult skull of *Hypacrosaurus stebingeri* (Ornithischia: Lambeosaurinae): anatomy and comparison. *Historical Biology* 23: 63-72.

Brink, K. S., Zelenitsky, D. K., Evans, D. C., Horner, J. R., and Therrien, F. 2014. Cranial morphology and variation in *Hypacrosaurus stebingeri* (Ornithischia: Hadrosauridae). In *Hadrosaurs* (eds. D. Evans and D. Eberth), pp. 245-265. Bloomington: Indiana University Press.

Brown, B. 1912. A crested dinosaur from the Edmonton Cretaceous. *Bulletin of the American Museum of Natural History* 31: 131-136.

Brown, B. 1913a. The skeleton of *Saurolophus*, a crested duck-billed dinosaur from the Edmonton Cretaceous. *Bulletin of the American Museum of Natural History* 32: 387-393.

Brown, B. 1913b. A new trachodont dinosaur, *Hypacrosaurus*, from the Edmonton Cretaceous of Alberta. *Bulletin of the American Museum of Natural History* 32: 395-406.

Brown, B. 1914. *Corythosaurus casuarius*, a new crested dinosaur from the Belly River Cretaceous, with provisional classification of the family Trachodontidae. *Bulletin of the American Museum of Natural History* 33: 559-564.

Brown, B. 1916. *Corythosaurus casuarius*: skeleton, musculature and epidermis. *Bulletin of the American Museum of Natural History* 35: 709-716.

Campione, N. E. and Evans, D. C. 2011. Cranial growth and variation in edmontosaurs (Dinosauria: Hadrosauridae): Implications for latest Cretaceous megaherbivore diversity in North America. *PLoS ONE* 6(9): e25186.

Campione, N. E. 2014. Postcranial anatomy of *Edmontosaurus regalis* (Hadrosauridae) from the Horseshoe Canyon Formation, Alberta, Canada. In *Hadrosaurs* (eds. D. Evans and D. Eberth), pp. 208-244. Bloomington: Indiana University Press.

Carpenter, K., Dilkes, D., and Weishampel, D. B. 1995. The dinosaurs of the Niobrara Chalk Formation (Upper Cretaceous, Kansas). *Journal of Vertebrate Paleontology* 15: 275-297.

Carpenter, K. and Ishida, Y. 2010. Early and “middle” Cretaceous iguanodonts in time and space. *Journal of Iberian Geology* 36: 145-164.

Cruzado-Caballero, P., Canudo, J. I., Moreno-Azanza, M., and Ruiz-Omeñaca, J. I. 2013. New material and phylogenetic position of *Arenysaurus ardevoli*, a lambeosaurine dinosaur from the late Maastrichtian of Arén (northern Spain). *Journal of Vertebrate Paleontology* 33: 1367-1384.

Cuthbertson, R. S. and Holmes, R. B. 2010. The first complete description of the holotype of *Brachylophosaurus canadensis* Sternberg, 1953 (Dinosauria: Hadrosauridae) with comments on intraspecific variation. *Zoological Journal of the Linnean Society* 159: 373-397.

Dalla Vecchia, F. M. 2009a. *Tethyshadros insularis*, a new hadrosauroid dinosaur (Ornithischia) from the Upper Cretaceous of Italy. *Journal of Vertebrate Paleontology* 29: 1100-1116.

Dalla Vecchia, F. M. 2009b. *Telmatosaurus* and the other hadrosauroids of the Cretaceous European Archipelago. An update. *Natura Nascosta* 39: 1-18.

Dilkes, D. W. 1999. Appendicular myology of the hadrosaurian dinosaur *Maiasaura peeblesorum* from the Late Cretaceous (Campanian) of Montana. *Transactions of the Royal Society of Edinburgh: Earth Sciences* 90: 87-125.

Evans, D. C. and Reisz, R. R. 2007. Anatomy and relationships of *Lambeosaurus magnicristatus*, a crested hadrosaurid dinosaur (Ornithischia) from the Dinosaur Park Formation, Alberta. *Journal of Vertebrate Paleontology* 27: 373-393.

Evans, D. C., Reisz, R. R., and DuPuis, K. 2007. A juvenile *Parasaurolophus* (Ornithischia: Hadrosauridae) braincase from Dinosaur Provincial Park, Alberta, with comments on crest ontogeny in the genus. *Journal of Vertebrate Paleontology* 27: 642-650.

Evans, D. C., Bavington, R., and Campione, N. E. 2009. An unusual hadrosaurid braincase from the Dinosaur Park Formation and the biostratigraphy of *Parasaurolophus* (Ornithischia: Lambeosaurinae) from southern Alberta. *Canadian Journal of Earth Sciences* 46: 791-800.

Evans, D. C. 2010. Cranial anatomy and systematics of *Hypacrosaurus altispinus*, and a comprehensive analysis of skull growth in lambeosaurine hadrosaurids (Dinosauria: Ornithischia). *Zoological Journal of the Linnean Society* 159: 398-434.

Freedman Fowler, E. A. and Horner, J. R. 2015. A new brachylophosaurin hadrosaur (Dinosauria: Ornithischia) with an intermediate nasal crest from the Campanian Judith River Formation of northcentral Montana. *PLoS ONE* 10(11): e0141304.

Gasca, J. M., Canudo, J. I., and Moreno-Azanza, M. 2014. On the diversity of Iberian iguanodont dinosaurs: new fossils from the lower Barremian, Teruel province, Spain. *Cretaceous Research* 50: 264-272.

Gasulla, J. M., Escaso, F., Narváez, I., Ortega, F., and Sanz, J. L. 2015. A new sail-backed styracosternan (Dinosauria: Ornithopoda) from the Early Cretaceous of Morella, Spain. *PLoS ONE* 10(12): e0144167.

Gates, T. A. and Sampson, S. D. 2007. A new species of *Gryposaurus* (Dinosauria:

Hadrosauridae) from the late Campanian Kaiparowits Formation, southern Utah, USA. *Zoological Journal of the Linnean Society* 151: 351-376.

Gates, T. A., Horner, J. R., Hanna, R. R., and Nelson, C. R. 2011. New unadorned hadrosaurine hadrosaurid (Dinosauria, Ornithopoda) from the Campanian of North America. *Journal of Vertebrate Paleontology* 31: 798-811.

Gates, T. A., Lund, E. K., Boyd, C. A., DeBlieux, D. D., Titus, A. L., Evans, D. C., Getty, M. A., Kirkland, J. I., and Eaton, J. G. 2013. Ornithopod dinosaurs from the Grand Staircase-Escalante National Monument region, Utah, and their role in paleobiogeographic and macroevolutionary studies. In *At the Top of the Grand Staircase: the Late Cretaceous of Southern Utah* (eds. A. L. Titus and M. A. Loewen), pp. 463-481. Bloomington: Indiana University Press.

Gates, T. A. and Scheetz, R. 2015. A new saurolophine hadrosaurid (Dinosauria: Ornithopoda) from the Campanian of Utah, North America. *Journal of Systematic Palaeontology* 13: 711-725.

Gates, T. A., Tsogtbaatar, K., Zanno, L. E., Chinzorig, T., and Watabe, M. 2018. A new iguanodontian (Dinosauria: Ornithopoda) from the Early Cretaceous of Mongolia. *PeerJ* 6: e5300.

Gilmore, C. W. 1909. Osteology of the Jurassic reptile *Camptosaurus*, with a revision of the species of the genus, and descriptions of two new species. *Proceedings of the United States National Museum* 36: 197-332.

Gilmore, C. W. 1924a. On the genus *Stephanosaurus*, with a description of the type specimen of *Lambeosaurus lambei*, Parks. *Canada Department of Mines (Geological Survey of Canada) Bulletin* 38: 29-48.

Gilmore, C. W. 1924b. On the skull and skeleton of *Hypacrosaurus*, a helmet-rested dinosaur from the Edmonton Cretaceous of Alberta. *Canada Department of Mines (Geological Survey of Canada) Bulletin* 38: 49-64.

Gilmore, C. W. 1933. On the dinosaurian fauna of the Iren Dabasu Formation. *Bulletin of the American Museum of Natural History* 67: 23-78.

Gilmore, C. W. and Stewart, D. R. 1945. A new sauropod dinosaur from the Upper Cretaceous of Missouri. *Journal of Paleontology* 19: 23-29.

Gilmore, C. W. 1945. *Parrosaurus*, n. name, replacing *Neosaurus* Gilmore, 1945. *Journal of Paleontology* 19: 540.

Godefroit, P., Dong Z.-M., Bultynck, P., Li H., and Feng L. 1998. New *Bactrosaurus* (Dinosauria: Hadrosauroidea) material from Iren Dabasu (Inner Mongolia, P. R. China). *Bulletin de l’Institut royal des Sciences naturelles de Belgique, Sciences de la Terra* 68 (Supplement): 3-70.

Godefroit, P., Zan S., and Jin L. 2001. The Maastrichtian (Late Cretaceous) lambeosaurine dinosaur *Charonosaurus jiayinensis* from north-eastern China. *Bulletin de l’Institut royal des Sciences naturelles de Belgique, Sciences de la Terre* 71: 119-168.

Godefroit, P., Alifanov, V., and Bolotsky, Y. 2004a. A re-appraisal of *Aralosaurus tuberiferus* (Dinosauria, Hadrosauridae) from the Late Cretaceous of Kazakhstan. *Bulletin de l’Institut royal des Sciences naturelles de Belgique, Sciences de la Terre* 74 (Supplement): 139-154.

Godefroit, P., Bolotsky, Y. L., and van Itterbeeck, J. 2004b. The lambeosaurine dinosaur *Amurosaurus riabinini*, from the Maastrichtian of Far Eastern Russia. *Acta Palaeontologica Polonica* 49: 585-618.

Godefroit, P., Codrea, V., and Weishampel, D. B. 2009. Osteology of *Zalmoxes shqiperorum*

(Dinosauria, Ornithopoda), based on new specimens from the Upper Cretaceous of Nǎlaţ-Vad (Romania). *Geodiversitas* 31: 525-553.

Godefroit, P., Escuillié, F., Bolotsky, Y. L., and Lauters, P. 2012a. A new basal hadrosauroid dinosaur from the Upper Cretaceous of Kazakhstan. In Bernissart Dinosaurs and Early Cretaceous Terrestrial Ecosystems (ed. P. Godefroit), pp. 334-358. Bloomington: Indiana University Press.

Godefroit, P., Bolotsky, Y. L., and Lauters, P. 2012b. A new saurolophine dinosaur from the latest Cretaceous of far eastern Russia. *PLoS ONE* 7(5): e36849.

Godefroit, P., Bolotsky, Y. L., and Bolotsky, I. Y. 2012c. Osteology and relationships of *Olorotitan arharensis*, a hollow-crested hadrosaurid dinosaur from the latest Cretaceous of Far Eastern Russia. *Acta Palaeontologica Polonica* 57: 527-560.

Head, J. J. 1998. A new species of basal hadrosaurid (Dinosauria, Ornithischia) from the Cenomanian of Texas. *Journal of Vertebrate Paleontology* 18: 718-738.

Head, J. J. 2001. A reanalysis of the phylogenetic position of *Eolambia caroljonesa* (Dinosauria, Iguanodontia). *Journal of Vertebrate Paleontology* 21: 392-396.

Hooley, R. W. 1925. On the skeleton of *Iguanodon atherfieldensis* sp. nov., from the Wealden Shales of Atherfield (Isle of Wight). *Quarterly Journal of the Geological Society of London* 81: 1-61.

Horner, J. R. 1983. Cranial osteology and morphology of the type specimen of *Maiasaura peeblesorum* (Ornithischia: Hadrosauridae), with discussion of its phylogenetic position. *Journal of Vertebrate Paleontology* 3: 29-38.

Horner, J. R. 1992. Cranial morphology of *Prosaurolophus* (Ornithischia: Hadrosauridae) with descriptions of two new hadrosaurid species and an evaluation of hadrosaurid phylogenetic relationships. *Museum of the Rockies Occasional Paper* 2: 1-119.

Horner, J. R., Weishampel, D. B., and Forster, C. A. 2004. Hadrosauridae. In *The Dinosauria:*

*Second Edition* (eds. D. B. Weishampel, P. Dodson, and H. Osmólska), pp. 438-463. Berkeley: University of California Press.

Hu C.-C. 1973. A new hadrosaur from the Cretaceous of Chucheng, Shantung. *Acta Geologica Sinica* 2: 179-206.

Hunt, A. P. and Lucas, S. G. 1993. Cretaceous vertebrates of New Mexico. *Vertebrate Paleontology in New Mexico, New Mexico Museum of Natural History and Science Bulletin* 2: 77-91.

Kirkland, J. I. 1998. A new hadrosaurid from the upper Cedar Mountain Formation (Albian-Cenomanian: Cretaceous) of eastern Utah - the oldest known hadrosaurid (lambeosaurine?). In *Lower and Middle Cretaceous Terrestrial Ecosystems* (eds. S. G. Lucas, J. I. Kirkland, and J. W. Estep). *New Mexico Museum of Natural History and Science Bulletin* 14: 283-295.

Kobayashi, Y. and Azuma, Y. 2003. A new iguanodontian (Dinosauria: Ornithopoda) from the Lower Cretaceous Kitadani Formation of Fukui Prefecture, Japan. *Journal of Vertebrate Paleontology* 23: 166-175.

Lambe, L. M. 1914. On *Gryposaurus notabilis*, a new genus and species of trachodont dinosaur from the Belly River Formation of Alberta, with a description of the skull of *Chasmosaurus belli*. *The Ottawa Naturalist* 27: 145-155.

Lambe, L. M. 1917. A new genus and species of crestless hadrosaur from the Edmonton Formation of Alberta. *The Ottawa Naturalist* 31: 65-73.

Langston, Jr., W. 1960. The vertebrate fauna of the Selma Formation of Alabama, part VI: the dinosaurs. *Fieldiana* 3: 315-361.

Lu, J. 1997. A new Iguanodontidae (*Probactrosaurus mazongshanensis* sp. nov.) from Mazongshan Area, Gansu Province, China. In *Sino-Japanese Silk Road Dinosaur Expedition* (ed. Z. Dong), pp. 27-47. Beijing: China Ocean Press.

Lucas, S. G., Spielmann, J. A., Sullivan, R. M., Hunt, A. P., and Gates, T. 2006. *Anasazisaurus*, a hadrosaurian dinosaur from the Upper Cretaceous of New Mexico. In *Late Cretaceous Vertebrates from the Western Interior* (eds. S. G. Lucas and R. M. Sullivan). *New Mexico Museum of Natural History and Science Bulletin* 35: 293-297.

Maryańska, T. and Osmólska, H. 1981. Cranial anatomy of *Saurolophus angustirostris* with comments on the Asian Hadrosauridae (Dinosauria). *Palaeontologia Polonica* 42: 5-24.

Maryańska, T. and Osmólska, H. 1984. Postcranial anatomy of *Saurolophus angustirostris* with comments on other hadrosaurs. *Palaeontologia Polonica* 46: 119-141.

McDonald, A. T., Kirkland, J. I., DeBlieux, D. D., Madsen, S. K., Cavin, J., Milner, A. R. C., and Panzarin, L. 2010a. New basal iguanodonts from the Cedar Mountain Formation of Utah and the evolution of thumb-spiked dinosaurs. *PLoS ONE* 5(11): e14075.

McDonald, A. T., Wolfe, D. G., and Kirkland, J. I. 2010b. A new basal hadrosauroid (Dinosauria: Ornithopoda) from the Turonian of New Mexico. *Journal of Vertebrate Paleontology* 30: 799-812.

McDonald, A. T. 2011. The taxonomy of species assigned to *Camptosaurus* (Dinosauria: Ornithopoda). *Zootaxa* 2783: 52-68.

McDonald, A. T. 2012. The status of *Dollodon* and other basal iguanodonts (Dinosauria: Ornithischia) from the Lower Cretaceous of Europe. *Cretaceous Research* 33: 1-6.

McDonald, A. T., Espílez, E., Mampel, L., Kirkland, J. I., and Alcalá, L. 2012a. An unusual new basal iguanodont (Dinosauria: Ornithopoda) from the Lower Cretaceous of Teruel, Spain. *Zootaxa* 3595: 61-76.

McDonald, A. T., Bird, J., Kirkland, J. I., and Dodson, P. 2012b. Osteology of the basal hadrosauroid *Eolambia caroljonesa* (Dinosauria: Ornithopoda) from the Cedar Mountain Formation of Utah. *PloS ONE* 7(10): e45712.

McDonald, A. T., Maidment, S. C. R., Barrett, P. M., You, H.-L., and Dodson, P. 2014. Osteology of the basal hadrosauroid *Equijubus normani* (Dinosauria, Ornithopoda) from the Early Cretaceous of China. In *Hadrosaurs* (eds. D. Evans and D. Eberth), pp. 44-72. Bloomington: Indiana University Press.

McDonald, A. T., Gates, T. A., Zanno, L. E., and Makovicky, P. J. 2017. Anatomy, taphonomy, and phylogenetic implications of a new specimen of *Eolambia caroljonesa* (Dinosauria: Ornithopoda) from the Cedar Mountain Formation, Utah, USA. *PLoS ONE* 12(5): e0176896.

McGarrity, C. T., Campione, N. E., and Evans, D. C. 2013. Cranial anatomy and variation in *Prosaurolophus maximus* (Dinosauria: Hadrosauridae). *Zoological Journal of the Linnean Society* 167: 531-568.

Mo J., Zhao Z., Wang W., and Xu X. 2007. The first hadrosaurid dinosaur from southern China. *Acta Geologica Sinica – English Edition* 81: 550-554.

Norman, D. B. 1980. On the ornithischian dinosaur *Iguanodon bernissartensis* from Belgium. *Mémoires Institut Royal des Sciences Naturelles de Belgique* 178: 1-103.

Norman, D. B. 1986. On the anatomy of *Iguanodon atherfieldensis* (Ornithischia: Ornithopoda). *Bulletin de l’Institut Royal des Sciences Naturelles de Belgique* 56: 281-372.

Norman, D. B. 1993. Gideon Mantell’s “Mantel-piece”: the earliest well-preserved ornithischian dinosaur. *Modern Geology* 18: 225-245.

Norman, D. B. 1998. On Asian ornithopods (Dinosauria: Ornithischia). 3. A new species of iguanodontid dinosaur. *Zoological Journal of the Linnean Society* 122: 291-348.

Norman, D. B. 2002. On Asian ornithopods (Dinosauria: Ornithischia). 4. *Probactrosaurus* Rozhdestvensky, 1966. *Zoological Journal of the Linnean Society* 136: 113-144.

Norman, D. B. 2004. Basal Iguanodontia. In *The Dinosauria: Second Edition* (eds. D. B. Weishampel, P. Dodson, and H. Osmólska), pp. 413-437. Berkeley: University of California Press.

Norman, D. B. 2010. A taxonomy of iguanodontians (Dinosauria: Ornithopoda) from the lower Wealden Group (Cretaceous: Valanginian) of southern England. *Zootaxa* 2489: 47-66.

Norman, D. B. 2011. On the osteology of the lower Wealden (Valanginian) ornithopod *Barilium dawsoni* (Iguanodontia: Styracosterna). *Special Papers in Palaeontology* 86: 165-194.

Norman, D. B. 2012. Iguanodontian taxa (Dinosauria: Ornithischia) from the Lower Cretaceous of England and Belgium. In Bernissart Dinosaurs and Early Cretaceous Terrestrial Ecosystems (ed. P. Godefroit), pp. 174-212. Bloomington: Indiana University Press.

Norman, D. B. 2014. On the history, osteology, and systematic position of the Wealden (Hastings Group) dinosaur *Hypselospinus fittoni* (Iguanodontia: Styracosterna). *Zoological Journal of the Linnean Society* 173: 92-189.

Ostrom, J. H. 1961. Cranial morphology of the hadrosaurian dinosaurs of North America. *Bulletin of the American Museum of Natural History* 122: 33-186.

Ostrom, J. H. 1963. *Parasaurolophus cyrtocristatus*, a crested hadrosaurian dinosaur from New Mexico. *Fieldiana, Geology* 14: 143-168.

Parks, W. A. 1920. The osteology of the trachodont dinosaur *Kritosaurus incurvimanus*. *University of Toronto Studies, Geological Series* 11: 1-75.

Parks, W. A. 1922. *Parasaurolophus walkeri*: a new genus and species of crested trachodont dinosaur. *University of Toronto Studies, Geological Series* 13: 1-32.

Parks, W. A. 1923. *Corythosaurus intermedius*: a new species of trachodont dinosaur. *University of Toronto Studies, Geological Series* 15: 1-57.

Parks, W. A. 1924. *Dyoplosaurus acutosquameus*, a new genus and species of armoured dinosaur; and notes on a skeleton of *Prosaurolophus maximus*. *University of Toronto Studies, Geological Series* 18: 26-36.

Pereda-Suberbiola, X., Canudo, J. I., Cruzado-Caballero, P., Barco, J. L., López-Martínez, N., Oms, O., and Ruiz-Omeñaca, J. I. 2009. The last hadrosaurid dinosaurs of Europe: a new lambeosaurine from the uppermost Cretaceous of Aren (Huesca, Spain). *Comptes Rendus Palevol* 8: 559-572.

Prieto-Márquez, A. 2005. New information on the cranium of *Brachylophosaurus canadensis* (Dinosauria, Hadrosauridae), with a revision of its phylogenetic position. *Journal of Vertebrate Paleontology* 25: 144-156.

Prieto-Márquez, A., Weishampel, D. B., and Horner, J. R. 2006a. The dinosaur *Hadrosaurus foulkii*, from the Campanian of the East Coast of North America, with a reevaluation of the genus. *Acta Palaeontologica Polonica* 51: 77-98.

Prieto-Márquez, A., Gaete, R., Rivas, G., Galobart, À., and Boada, M. 2006b. Hadrosauroid dinosaurs from the Late Cretaceous of Spain: *Pararhabdodon isonensis* revisited and *Koutalisaurus kohlerorum*, gen. et sp. nov. *Journal of Vertebrate Paleontology* 26: 929-943.

Prieto-Márquez, A. 2007. Postcranial osteology of the hadrosaurid dinosaur *Brachylophosaurus canadensis* from the Late Cretaceous of Montana. In *Horns and Beaks: Ceratopsian and Ornithopod Dinosaurs* (ed. K. Carpenter), pp. 91-115. Bloomington: Indiana University Press.

Prieto-Márquez, A. and Wagner, J. R. 2009. *Pararhabdodon isonensis* and *Tsintaosaurus spinorhinus*: a new clade of lambeosaurine hadrosaurids from Eurasia. *Cretaceous Research* 30: 1238-1246.

Prieto-Márquez, A. 2010a. The braincase and skull roof of *Gryposaurus notabilis* (Dinosauria, Hadrosauridae), with a taxonomic revision of the genus. *Journal of Vertebrate Paleontology* 30: 838-854.

Prieto-Márquez, A. 2010b. Global phylogeny of hadrosauridae (Dinosauria: Ornithopoda) using parsimony and Bayesian methods. *Zoological Journal of the Linnean Society* 159: 435-502.

Prieto-Márquez, A. and Norell, M. A. 2010. Anatomy and relationships of *Gilmoreosaurus mongoliensis* (Dinosauria: Hadrosauroidea) from the Late Cretaceous of Central Asia. *American Museum Novitates* 3694: 1-49.

Prieto-Márquez, A. 2011. Revised diagnoses of *Hadrosaurus foulkii* Leidy 1858 (the type genus and species of Hadrosauridae Cope, 1869) and *Claosaurus agilis* Marsh, 1872 (Dinosauria: Ornithopoda) from the Late Cretaceous of North America. *Zootaxa* 2765: 61-68.

Prieto-Márquez, A. 2012. The skull and appendicular skeleton of *Gryposaurus latidens*, a saurolophine hadrosaurid (Dinosauria: Ornithopoda) from the early Campanian (Cretaceous) of Montana, USA. *Canadian Journal of Earth Sciences* 49: 510-532.

Prieto-Márquez, A. and Wagner, J. R. 2013a. A new species of saurolophine hadrosaurid dinosaur from the Late Cretaceous of the Pacific coast of North America. *Acta Palaeontologica Polonica* 58: 255-268.

Prieto-Márquez, A. and Wagner, J. R. 2013b. The ‘unicorn’ dinosaur that wasn’t: a new reconstruction of the crest of *Tsintaosaurus* and the early evolution of the lambeosaurine crest and rostrum. *PLoS ONE* 8(11): e82268.

Prieto-Márquez, A., Dalla Vecchia, F. M., Gaete, R., and Galobart, A. 2013. Diversity, relationships, and biogeography of the lambeosaurine dinosaurs from the European Archipelago, with description of the new aralosaurin *Canardia garonnensis*. *PLoS ONE* 8(7): e69835.

Prieto-Márquez, A. 2014a. Skeletal morphology of *Kritosaurus navajovius* (Dinosauria: Hadrosauridae) from the Late Cretaceous of the North American south-west, with an evaluation of the phylogenetic systematics and biogeography of Kritosaurini. *Journal of Systematic Palaeontology* 12: 133-175.

Prieto-Márquez, A. 2014b. A juvenile *Edmontosaurus* from the late Maastrichtian (Cretaceous) of North America: Implications for ontogeny and phylogenetic inference in saurolophine dinosaurs. *Cretaceous Research* 50: 282-303.

Prieto-Márquez, A., Wagner, J. R., Bell, P. R., and Chiappe, L. M. 2015. The late-survifing ‘duck-billed’ dinosaur *Augustynolophus* from the upper Maastricthian of western North America and crest evolution in Saurolophini. *Geological Magazine* 152: 225-241.

Prieto-Márquez, A., Erickson, G. M., and Ebersole, J. A. 2016a. A primitive hadrosaurid from southeastern North America and the origin and early evolution of ‘duck-billed’ dinosaurs. *Journal of Vertebrate Paleontology*. DOI: 10.1080/02724634.2015.1054495

Prieto-Márquez, A., Erickson, G. M., and Ebersole, J. A. 2016b. Anatomy and osteohistology of the basal hadrosaurid dinosaur *Eotrachodon* from the uppermost Santonian (Cretaceous) of southern Appalachia. *PeerJ* 4: e1872.

Ramírez-Velasco, A. A., Benammi, M., Prieto-Márquez, A., Ortega, J. A., and Hernández-Rivera, R. 2012. *Huehuecanauhtlus tiquichensis*, a new hadrosauroid dinosaur (Ornithischia: Ornithopoda) from the Santonian (Late Cretaceous) of Michoacán, Mexico. *Canadian Journal of Earth Sciences* 49: 379-395.

Shibata, M. and Azuma, Y. 2015. New basal hadrosauroid (Dinosauria: Ornithopoda) from the Lower Cretaceous Kitadani Formation, Fukui, central Japan. *Zootaxa* 3914: 421-440.

Shibata, M., Jintasakul, P., Azuma, Y., and You, H.-L. 2015. A new basal hadrosauroid dinosaur from the Lower Cretaceous Khok Kruat Formation in Nakhon Ratchasima Province, northeastern Thailand. *PLoS ONE* 10(12): e0145904.

Sues, H.-D. and Averianov, A. 2009. A new basal hadrosauroid dinosaur from the Late Cretaceous of Uzbekistan and the early radiation of duck–billed dinosaurs. *Proceedings of the Royal Society B* 276: 2549-2555.

Sullivan, R. M. and Williamson, T. E. 1999. A new skull of *Parasaurolophus* (Dinosauria: Hadrosauridae) from the Kirtland Formation of New Mexico and a revision of the genus. *New Mexico Museum of Natural History and Science Bulletin* 15: 1-52.

Sullivan, R. M., Jasinski, S. E., Guenther, M., and Lucas, S. G. 2011. The first lambeosaurin (Dinosauria, Hadrosauridae, Lambeosaurinae) from the Upper Cretaceous Ojo Alamo Formation (Naashoibito Member), San Juan Basin, New Mexico. *Fossil Record 3, New Mexico Museum of Natural History and Science Bulletin* 53: 405-417.

Suzuki, D., Weishampel, D. B., and Minoura, N. 2004. *Nipponosaurus sachalinensis* (Dinosauria; Ornithopoda): anatomy and systematic position within Hadrosauridae. *Journal of Vertebrate Paleontology* 24: 145-164.

Taquet, P. 1976. Ostéologie d’*Ouranosaurus nigeriensis*, Iguanodontide du Crétacé Inférieur du Niger. *Géologie et Paléontologie du Gisement de Gadoufaoua (Aptien du Niger)*, Chapitre III. pp. 57-168.

Taquet, P. and Russell, D. A. 1999. A massively-constructed iguanodont from Gadoufaoua, Lower Cretaceous of Niger. *Annales de Paléontologie* 85: 85-96.

Trexler, D. L. 1995. A detailed description of newly-discovered remains of *Maiasaura*

*peeblesorum* (Reptilia: Ornithischia) and a revised diagnosis of the genus. MSc. Thesis, University of Calgary.

Tsogtbaatar, K., Weishampel, D. B., Evans, D. C., and Watabe, M. 2014. A new hadrosauroid (*Plesiohadros djadokhtaensis*) from the Late Cretaceous Djadokhtan fauna of southern Mongolia. In *Hadrosaurs* (eds. D. Evans and D. Eberth), pp. 108-135. Bloomington: Indiana University Press.

Tsogtbaatar, K., Weishampel, D. B., Evans, D. C., and Watabe, M. 2019. A new hadrosauroid (Dinosauria: Ornithopoda) from the Late Cretaceous Baynshire Formation of the Gobi Desert (Mongolia). *PLoS ONE* 14(4): e0208480.

Verdú, F. J., Royo-Torres, R., Cobos, A., and Alcalá, L. 2015. Perinates of a new species of *Iguanodon* (Ornithischia: Ornithopoda) from the lower Barremian of Galve (Teruel, Spain). *Cretaceous Research* 56: 250-264.

Vidarte, C. F., Calvo, M. M., Fuentes, F. M., and Fuentes, M. M. 2016. Un nuevo dinosaurio estiracosterno (Ornithopoda: Ankylopollexia) del Cretácico Inferior de España. *Spanish Journal of Palaeontology* 31: 407-446.

Wang, R.-F., You, H.-L., Xu, S.-C., Wang, S.-Z., Yi, J., Xie, L.-J., Jia, L., and Li, Y.-X. 2013. A new hadrosauroid dinosaur from the early Late Cretaceous of Shanxi Province, China. *PLoS ONE* 8(10): e77058.

Wang, X., Pan, R., Butler, R. J., and Barrett, P. M. 2011. The postcranial skeleton of the iguanodontian ornithopod *Jinzhousaurus yangi* from the Lower Cretaceous Yixian Formation of western Liaoning, China. *Earth and Environmental Science Transactions of the Royal Society of Edinburgh* 101: 135-159.

Weishampel, D. B. and Bjork, P. R. 1989. The first indisputable remains of *Iguanodon* (Ornithischia: Ornithopoda) from North America: *Iguanodon lakotaensis*, sp. nov. *Journal of Vertebrate Paleontology* 9: 56-66.

Weishampel, D. B., Norman, D. B., and Grigorescu, D. 1993. *Telmatosaurus transsylvanicus* from the Late Cretaceous of Romania: the most basal hadrosaurid dinosaur. *Palaeontology* 36: 361-385.

Weishampel, D. B., Jianu, C.-M., Csiki, Z., and Norman, D. B. 2003. Osteology and phylogeny of *Zalmoxes* (n. g.), an unusual euornithopod dinosaur from the latest Cretaceous of Romania. *Journal of Systematic Palaeontology* 1: 65-123.

Wiman, C. 1929. Die Kreide-dinosaurier aus Shantung. *Palaeontologia Sinica* 6: 1-63.

Wiman, C. 1931. *Parasaurolophus tubicen* n. sp. aus der Kreide in New Mexico*. Nova Acta Regiae Societatis Scientiarum Upsaliensis, Series IV* 7: 1-11.

Winkler, D. A., Murry, P. A., and Jacobs, L. L. 1997. A new species of *Tenontosaurus*

(Dinosauria: Ornithopoda) from the Early Cretaceous of Texas. *Journal of Vertebrate Paleontology* 17: 330­­-348.

Wu W. and Godefroit, P. 2012. Anatomy and relationships of *Bolong yixianensis*, and Early Cretaceous iguanodontoid dinosaur from western Liaoning, China. In Bernissart Dinosaurs and Early Cretaceous Terrestrial Ecosystems (ed. P. Godefroit), pp. 292-333. Bloomington: Indiana University Press.

Xing, H., Wang, D., Han, F., Sullivan, C., Ma, Q., He, Y., Hone, D. W. E., Yan, R., Du, F., and Xu, X. 2014a. A new basal hadrosauroid dinosaur (Dinosauria: Ornithopoda) with transitional features from the Late Cretaceous of Henan Province, China. *PLoS ONE* 9(6): e98821.

Xing H., Zhao X., Wang K., Li D., Chen S., Mallon, J. C., Zhang Y., and Xu X. 2014b. Comparative osteology and phylogenetic relationship of *Edmontosaurus* and *Shantungosaurus* (Dinosauria: Hadrosauridae) from the Upper Cretaceous of North America and East Asia. *Acta Geologica Sinica (English Edition)* 88: 1801-1840.

Xing, H., Mallon, J. C., and Currie, M. L. 2017. Supplementary cranial description of the types of *Edmontosaurus regalis* (Ornithischia: Hadrosauridae), with comments on the phylogenetics and biogeography of Hadrosaurinae. *PLoS ONE* 12(4): e0175253.

Xu X., Zhao X. J., Lu J.-C., Huang W.-B., Li Z.-Y., and Dong Z.-M. 2000. A new iguanodontian from Sangping Formation of Neixiang, Henan and its stratigraphical implications. *Vertebrata PalAsiatica* 38: 176-191.

Xu X., Tan Q., Gao Y., Bao Z., Yin Z., Guo B., Wang J., Tan L., Zhang Y., and Xing H. 2018. A large-sized basal ankylopollexian from East Asia, shedding light on early biogeographic history of Iguanodontia. *Science Bulletin* 63: 556-563.

You, H., Luo, Z., Shubin, N. H., Witmer, L. M., Tang, Z., and Tang, F. 2003a. The earliest-known duck-billed dinosaur from deposits of late Early Cretaceous age in northwest China and hadrosaur evolution. *Cretaceous Research* 24: 347-355.

You H., Ji Q., Li J., and Li Y. 2003b. A new hadrosauroid dinosaur from the mid-Cretaceous of Liaoning, China. *Acta Geologica Sinica-English Edition* 77: 148-154.

You H., Ji Q., and Li D. 2005. *Lanzhousaurus magnidens* gen. et sp. nov. from Gansu Province, China: the largest-toothed herbivorous dinosaur in the world. *Geological Bulletin of China* 24: 785-794.

You H.-L. and Li D.-Q. 2009. A new basal hadrosauriform dinosaur (Ornithischia: Iguanodontia) from the Early Cretaceous of northwestern China. *Canadian Journal of Earth Sciences* 46: 949-957.

You H., Li D., and Liu W. 2011. A new hadrosauriform dinosaur from the Early Cretaceous of Gansu Province, China. *Acta Geologica Sinica-English Edition* 85: 51-57.

You, H.-L., Li, D.-Q., and Dodson, P. 2014. *Gongpoquansaurus mazongshanensis* (Lü, 1997) comb. nov. (Ornithischia: Hadrosauroidea) from the Early Cretaceous of Gansu Province, northwestern China. In *Hadrosaurs* (eds. D. Evans and D. Eberth), pp. 73-76. Bloomington: Indiana University Press.

Zheng, W., Jin, X., Shibata, M., and Azuma, Y. 2014. An early juvenile specimen of *Bolong yixianensis* (Ornithopoda: Iguanodontia) from the Lower Cretaceous of Ningcheng County, Nei Mongol, China. *Historical Biology* 26: 236-251.
